# Searching for new plastic-degrading enzymes from the plastisphere of alpine soils using a metagenomic mining approach

**DOI:** 10.1371/journal.pone.0300503

**Published:** 2024-04-05

**Authors:** Beat Frey, Margherita Aiesi, Basil M. Rast, Joel Rüthi, Jérôme Julmi, Beat Stierli, Weihong Qi, Ivano Brunner

**Affiliations:** 1 Swiss Federal Institute for Forest, Forest Soils and Biogeochemistry, Snow and Landscape Research WSL, Birmensdorf, Switzerland; 2 Facoltà de Science Agrarie e Alimentari, University Degli Studi di Milano, Milano, Italy; 3 Functional Genomics Center Zürich, ETH Zürich and University of Zürich, Zürich, Switzerland; 4 Swiss Institute of Bioinformatics SIB, Geneva, Switzerland; St. Pius X College, Rajapuram, Kasaragod-Kannur University, INDIA

## Abstract

Plastic materials, including microplastics, accumulate in all types of ecosystems, even in remote and cold environments such as the European Alps. This pollution poses a risk for the environment and humans and needs to be addressed. Using shotgun DNA metagenomics of soils collected in the eastern Swiss Alps at about 3,000 m a.s.l., we identified genes and their proteins that potentially can degrade plastics. We screened the metagenomes of the plastisphere and the bulk soil with a differential abundance analysis, conducted similarity-based screening with specific databases dedicated to putative plastic-degrading genes, and selected those genes with a high probability of signal peptides for extracellular export and a high confidence for functional domains. This procedure resulted in a final list of nine candidate genes. The lengths of the predicted proteins were between 425 and 845 amino acids, and the predicted genera producing these proteins belonged mainly to *Caballeronia* and *Bradyrhizobium*. We applied functional validation, using heterologous expression followed by enzymatic assays of the supernatant. Five of the nine proteins tested showed significantly increased activities when we used an esterase assay, and one of these five proteins from candidate genes, a hydrolase-type esterase, clearly had the highest activity, by more than double. We performed the fluorescence assays for plastic degradation of the plastic types BI-OPL and ecovio^®^ only with proteins from the five candidate genes that were positively active in the esterase assay, but like the negative controls, these did not show any significantly increased activity. In contrast, the activity of the positive control, which contained a PLA-degrading gene insert known from the literature, was more than 20 times higher than that of the negative controls. These findings suggest that *in silico* screening followed by functional validation is suitable for finding new plastic-degrading enzymes. Although we only found one new esterase enzyme, our approach has the potential to be applied to any type of soil and to plastics in various ecosystems to search rapidly and efficiently for new plastic-degrading enzymes.

## Introduction

Synthetic and fossil-fuel-based polymers were developed in the late 1930s and quickly found their way into a variety of applications in industries and society. Because of their stability and durability, they have become indispensable in our daily lives. Today, about 390 million tons of plastic is produced worldwide every year [1]. The main types of fossil-fuel-based polymers that are produced are polyethylene (PE), polypropylene (PP), polyvinylchloride (PVC), polyurethane (PUR), polyethylene terephthalate (PET), and polystyrene (PS). In Europe, about 10% of the plastic production is circular, and about 2% involves bio-based or bio-attributed plastics [1]. Bio-based polymers include natural rubber (NR), polybutylene adipate terephthalate (PBAT), polylactic acid (PLA), and polycaprolactone (PCL), which are aliphatic polyesters [2]. Furthermore, starch and cellulose blends are among the main biopolymers.

In general, plastic materials are not biologically or chemically degradable, but they are weathered by physical factors, such as mechanical forces or UV light. Consequently, plastics accumulate in our environment, and in the long term they mainly remain as microplastics or nanoplastics [3]. Plastic particles, in particular microplastics, have appeared in food chains and are found in living organisms, causing certain harm [4,5].

Researchers in microbiology have investigated if and to what extent microorganisms can decompose plastics through enzymatic pathways [6,7]. Up to now, only a few enzymes have been found to act on low-density and low-crystalline (amorphous) PET and ester-based PUR. However, the identification of plastic-active enzymes–to understand their potential role in nature or to use them in biotechnological processes–is an emerging research field, and the application of these enzymes is still at its beginning [5].

There are many ways to find microbial enzymes that are potentially active in plastic degradation [8–10]. The most recently applied and innovative way to find an enzymatic machinery capable of plastic degradation is through metagenomics or metatranscriptomics [11]. While shotgun metagenomics extracts DNA from all cells in a community and then cuts it into tiny fragments that are sequenced independently [12], metatranscriptomics provides information about genes expressed by the community as a whole [13]. One of the major advantages of metagenomics is that it provides access to genetic information about the non-culturable microbes that still have not been functionally characterized [14]. Considering that less than 1% of the total microbiota can be cultured in the laboratory [15], the large proportion that is left represents an unexplored treasure with high functional potential. However, while the genome-resolved information about microbial communities from nearly every environment on earth is available, the ability to screen the metagenome for direct enzyme discovery remains challenging. Inferring the function of a new enzyme directly from sequencing data is like searching for a needle in a haystack. For this reason, the development of computational and experimental strategies to mine metagenomes is playing an increasingly important role in research [16].

In the present study we performed enzyme searching directly from shotgun metagenomic data. Other methodologies that have been applied over the years rely on activity-guided or polymerase chain reaction (PCR)-based functional metagenomics [16]. The main difference is that direct metagenomic screening relies on the untargeted sequencing of the full amount of DNA coming from samples, while activity-guided and PCR-based functional metagenomics start with a pre-screening step to decrease the amount of DNA to sequence, analyze and test. Moreover, the latter two applications do not give taxonomic information, and they are limited to types of reactions that can be screened rapidly [16]. Compared with activity- and PCR-based functional metagenomics, studies in which enzymes have been discovered from direct metagenome sequencing are still rare (e.g. [17]). The roadmap for metagenomic enzyme searching includes three phases: (1) *in silico* screening of a metagenome, leading to the identification of protein sequences of candidate genes; (2) cloning of corresponding genes into a suitable host and heterologous expression; and (3) activity testing and biochemical characterization of expressed enzymes.

According to Robinson et al. [16], the advantages of shotgun metagenomic sequences are that they provide a complete functional profile, as well as a genomic context and taxonomy, obtained through binning and assembly. Furthermore, *in silico* methods for enzyme searching can be advantageous in phylogenetics, in sequence similarity networking, in three-dimensional (3D) structural predictions, in the analysis of motifs and active site architecture, and in machine learning based on algorithms for protein function prediction–to identify hidden relationships between protein sequences, structures and functions [16]. Recently, Sonnendecker et al. [18] discovered a polyester hydrolase that was isolated from a compost metagenome.

In this study we applied shotgun metagenomics with the aim of finding new plastic-degrading enzymes from the plastisphere of aliphatic polyesters of poorly explored cold environments, i.e. alpine soils. The term “plastisphere” was introduced and characterized by Zettler et al. [19] and Amaral-Zettler [20] and has since been a central topic of microbial research on plastic pollution [21]. The plastisphere, which comprises the microbial community on plastic debris, can be considered an analogue of the rhizosphere and refers to an environment including the microbial community and not just the microbial community inhabiting that environment [21].

Recently, we discovered plastic-degrading microbial strains growing on the plastisphere [22–24]. Furthermore, we found that α/β-hydrolase genes were enriched in the plastisphere of two biodegradable plastic types used as mulching films, BI-OPL and ecovio^®^, when we investigated the microbial genetic potential of plastic degradation using shotgun metagenomics in cryospheric soils from a high-alpine site in the Swiss Alps [25]. Both types of plastic contain PBAT and PLA, with both polymers becoming increasingly important in both agriculture and packaging [26]. The microbial communities of the cryospheric soils at this site have been characterized previously [27–30]. In the present study, we specifically aimed to: (a) compare the outcome of shotgun DNA metagenomics of plastisphere soil with that of bulk soil, (b) synthesize and clone candidate genes that are highly abundant in the plastisphere and are potentially active in plastic degradation, (c) express the cloned genes, and (d) test the proteins produced from these genes for enzymatic activity using functional assays that represent plastic degradation.

## Materials and methods

### Site description and soil sampling

Alpine soil was collected from 0 to 10 cm depth on the north-exposed slope of Muot da Barba Peider in the eastern Swiss Alps, at 2,979 m a.s.l. The Swiss Federal Institute for Forest, Snow and Landscape Research WSL has a general permit from the community of Pontresina to use the surrounding area for scientific purposes. The mean annual soil temperature at a depth of 5 cm is about -1.5°C, with a range of -13°C to 23°C, and the mean annual precipitation is around 1,500 mm. The bedrock consists of gneiss from the upper Austroalpine Languard nappe, and the soil has a pH of 6.5. The soil is 80% sand, 16% silt and 4% clay, and it has a carbon content of 0.14% and a nitrogen content below the detection limit. For a detailed description of the soil physico-chemical characteristics, see also Frey et al. [21] and Perez-Mon et al. [30]. Soils were sampled in September 2019 at three independent field locations at least 2 m from each other but representing the same soil type. Soils were then transported in cold boxes to the laboratory and stored in closed plastic bags at 4°C in the dark until the incubation experiments.

### Plastic film incubation and DNA extraction

The incubation experiments began in January 2020. The stored soil samples were transferred to nine autoclavable polycarbonate plant culture Magenta boxes (Magenta GA-7; Sigma-Aldrich, St. Louis, USA), each of which contained about 180 g of soil. This resulted in three Magenta boxes left untreated as controls (bulk soil), three that were incubated with BI-OPL (Oerlemans Plastic, Genderen, Netherlands) plastic film pieces (6×6 cm) and three that were incubated with ecovio^®^ (BASF Ludwigshafen, Germany) plastic film pieces (6×6 cm). BI-OPL contains about 61% PBAT and 13% PLA, while ecovio^®^ contains about 64% PBAT and 3% PLA [24]. Four pieces of plastic film were buried in the soil for each Magenta box. Before incubation, the boxes were autoclaved and the plastic films were sterilized in 70% ethanol and washed in sterile Milli-Q water. The boxes were then covered with lids that had a central opening that was covered with a sterile gauze pad, to allow gas exchange. They were then incubated at 15°C in the dark for a period of 5 months. The moisture content was monitored gravimetrically and adjusted weekly by adding sterile dH_2_O.

At the end of the incubation experiment, about 650 mg of soil was taken from the middle of the control boxes (bulk soil) and filled into DNA extraction tubes. For the Magenta boxes incubated with the plastic films (plastisphere samples), small soil particles adhering to the plastic films were manually scraped off and transferred into the DNA extraction tubes. In addition, a 1×2 mm section was cut from each of the four plastic film pieces per Magenta box and added to these extraction tubes, for a total fresh weight of approximately 650 mg. Samples were frozen at -20°C until DNA extraction was performed. DNA was extracted using the DNeasy PowerSoil Pro Kit (Qiagen, Hilden, Germany) according to the manufacturer’s protocol and quantified using the Qubit™ high-sensitivity assay for double-stranded DNA (Thermo Fisher Scientific, Waltham, MA, USA; for details see [25]).

### Shotgun DNA sequencing and data processing

TruSeq® DNA library preparation and Illumina NextSeq v2.5 shotgun sequencing were performed by Microsynth AG (Balgach, Switzerland) to obtain paired end raw reads of 150 bp. A customized pipeline was then used to pre-process the raw reads, to assemble the reads into contigs, to annotate the contigs for functionality and taxonomy, and to analyze the abundance of protein-coding genes [25,31]. Briefly, Trimmomatic v0.36 [32] was used to trim and filter the raw reads, MEGAHIT v1.2.9 [33] was used to assemble the pre-processed read pairs into contigs (>200 bp), MetaGeneMark v3.38 [34] was used to predict amino acid coding sequences of proteins in the contigs, and SWORD v1.0.3 [35] was used to annotate these predicted genes against databases for functional annotation. Taxonomic assignment of the genes was performed by applying the Kaiju v1.7.4 program [36]. For more detailed information, see Rüthi et al. [25].

### Candidate gene selection

The metagenomic output was progressively analyzed to identify putative plastic degraders. Differential abundance analysis was performed to compare protein-coding genes from the plastisphere with those extracted from the bulk soil, calculating the logarithmic fold change (log_2_-fold change) for each gene between the two microbial niches, with field replicates pooled for analysis [23]. The log_2_-fold change was calculated using DEseq2 v1.26.0 [37], which provides a method to test for differential expression using negative binomial generalized linear models.

The 1,000 genes with the highest log_2_-fold change were then considered in greater detail. To narrow down the group of candidate genes, initial broad filtering was performed by screening the metagenomic annotation. This step involved isolating genes associated with the functions known in the literature to be responsible for plastic degradation, such as cutinases, esterases, depolymerases, lipases and serine proteases [38–41].

Candidate genes were functionally annotated against a set of databases with the command-line tool InterProScan 5.32–71 (https://interproscan-docs.readthedocs.io/en/latest/Citing.html) [42]. The annotations for the following databases were considered: eggNOG provides orthology relationships, functional annotation, and gene evolutionary histories [43]; Gene3D provides protein domain annotations for sequences from the major sequence databases [44]; Pfam enables searches for the sequences of interest against a comprehensive collection of protein domains and families [45]; PMDB (Protein Model Database) stores manually built 3D protein models and provides access to models published in the scientific literature [46]; and SUPERFAMILY provides structural and functional annotation for all proteins and genomes [47]. In addition, Foldseek has been used for fast and accurate protein structure searches and sensitive comparison of large structural sets [48].

NCBI-blastp (https://blast.ncbi.nlm.nih.gov/Blast.cgi?PAGE=Proteins) was applied to compare protein queries with protein data based on local alignment (searched in September 2023).

### Analysis of structural elements of predicted proteins

The SignalP v5.0 server (https://services.healthtech.dtu.dk/services/SignalP-5.0/) was used to predict the presence of signal peptides and the location of their cleavage sites in proteins from Archaea, gram-positive and gram-negative Bacteria, and Eukarya. In Bacteria and Archaea, SignalP can discriminate between three types of signal peptides: (1) “standard” secretory signal peptides transported by the Sec translocon and cleaved by signal peptidase I (Sec/SPI), (2) lipoprotein signal peptides transported by the Sec translocon and cleaved by signal peptidase II (Sec/SPII), and (3) Tat signal peptides transported by the Tat translocon and cleaved by signal peptidase I (Tat/SPI). SignalP predicts putative signal peptides by linking each gene to the probability of the presence of a secretion signal peptide, its position, and the type of signal. Additionally, the server provides information about the cleavage site of the signal peptide and its probability. Only candidates with a signal peptide presence probability > 80% were selected. To predict transmembrane domains in proteins, the TMHMM Server v2.0 was used (https://services.healthtech.dtu.dk/services/TMHMM-2.0/). Since transmembrane domains can interfere with the recovery of soluble proteins during *Escherichia coli* expression, the prediction of its presence can be important.

The 3D structure of the proteins was predicted from the amino acid sequence with the AlphaFold2 program [49] using MMseqs2 and ColabFold v1.5.3 (https://colab.research.google.com/github/sokrypton/ColabFold/blob/main/AlphaFold2.ipynb) [50]. The obtained 3D structures were analyzed with the P2Rank software (https://prankweb.cz/), a machine-learning-based method for predicting ligand binding sites from protein structure [51–54]. P2Rank gives information on pocket score, probability, amino acids involved, and conservation.

The “function prediction” tool from the Plastics Microbial Biodegradation Database (PMBD; http://pmbd.genome-mining.cn/predict/), a collection of information on the microbial biodegradation of plastics, was used to analyze the protein sequences [55]. PMBD contains a total of 949 microorganism–plastic relationships and 79 genes confirmed through literature searches, as well as more than 8,000 automatically annotated enzyme sequences from the TrEMBL section of the UniProt database that are thought to be involved in the biodegradation of plastics [55].

**Fig 1:**
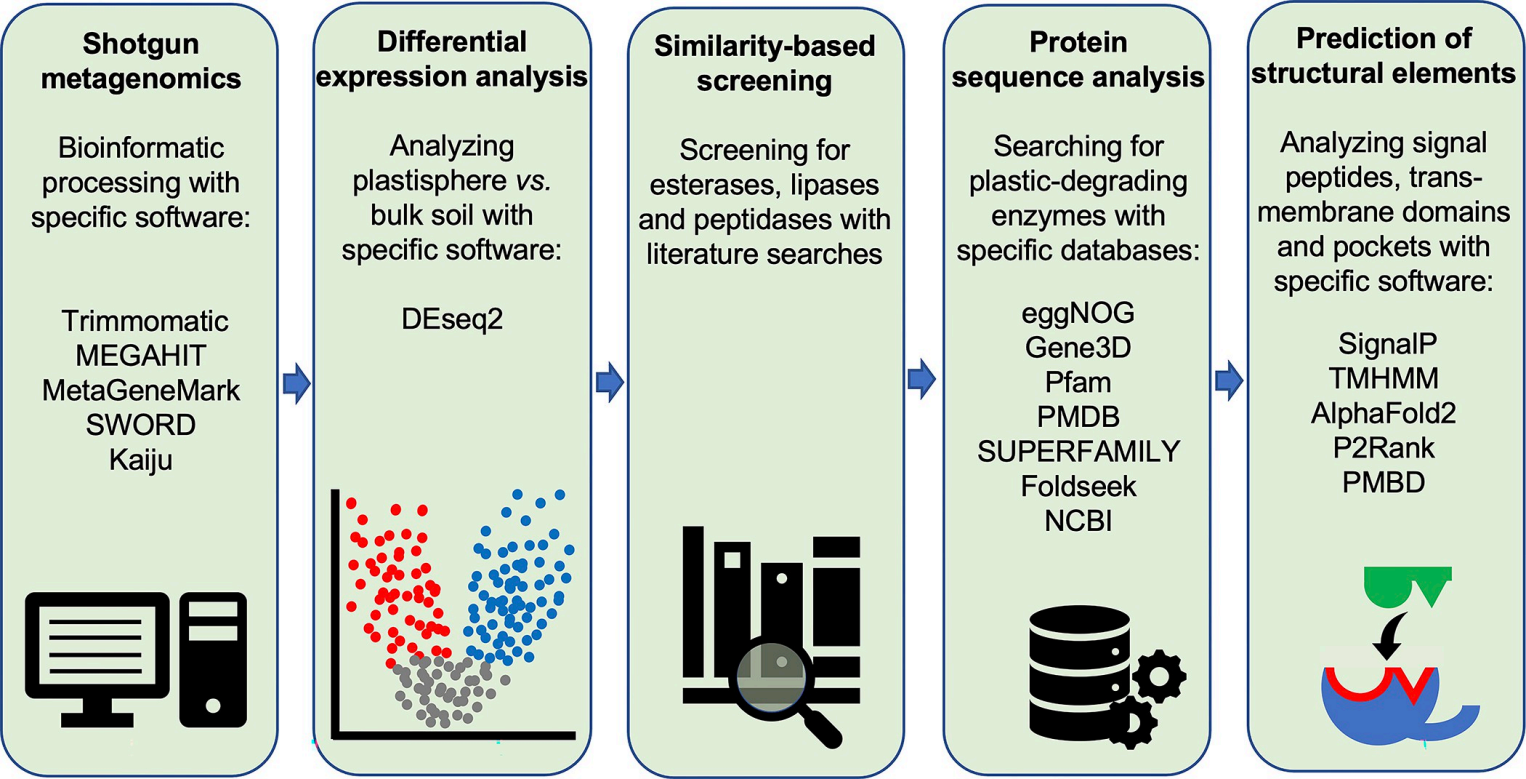
Workflow for the selection of candidate genes. For abbreviations see the Materials and Methods section.

An overview of the workflow for the selection of candidate genes is shown in **Fig 1**.

### Transformation of candidate genes

The nucleotide sequences were synthesized and cloned into the pMAL-p5G expression vector from GenScript® (EU Headquarter, Rijswijk, Netherlands; **S1 Fig**) with Nde I and EcoR V chosen as the cloning site. The vector pMAL-p5G is designed to produce maltose-binding protein (MBP) fusions in the cytoplasm, and the effectiveness of MBP as a solubility enhancer tag has been shown previously [56,57]. The genes were codon optimized for expression with *Escherichia coli* BL21(DE3)pLysS (Promega AG, Dübendorf, Switzerland) as the host strain, where the stop codon was omitted. The optimal nucleic acid sequences were predicted using the OptimumGeneTM algorithm (GenScript®), which optimizes a variety of parameters that are critical for the efficiency of gene expression. The Codon Adaptation Index (CAI) and the Frequency of Optimal Codons (FOP) were used as indicators for the goodness of gene expression. Generation of the constructs, optimization of nucleotide sequences, and quality control were performed with GenScript®. Vector constructs were delivered as lyophilized powder (4 μg for each construct). For transformation, lyophilized DNA sample plasmids (4 μg) were centrifuged (6,000 g for 1 min, 4°C), dissolved in 40 μL milli-Q water, and vortexed for 1 min to create the stock solution (0.1 μg μL^-1^), which was stored at -20°C. The stock solution was then diluted to 1:10 to obtain a 10 ng μL^-1^ solution, which was stored at 4°C. The *E*. *coli* strain BL21(DE3)pLysS was selected as the host strain for the pMAL-p5G vector. The inserted sequences were deprived of the native signal sequence, which was substituted with pelB for periplasmic secretion. pMAL-p5G is designed to produce maltose-binding protein (MBP) fusions, where the protein of interest can be cleaved from the MBP with the specific protease Genenase™1.

For the transformation procedure, sterile 1.5 mL Eppendorf tubes were first chilled on ice. In each tube, 2 μL of plasmid (20 ng) was then mixed with 20 μL of competent cells of *E*. *coli* strain BL21(DE3)pLysS that had been previously thawed on ice. After a 30-min incubation on ice, heat-shocking was performed at 42°C for 45 seconds. Subsequently, 500 μL of super optimal broth with catabolite repression (SOC)-medium was added to each transformation reaction and a second incubation was carried out for 1 h at 37°C with shaking (270 rpm). For each transformation reaction, 50 μL and 150 μL of the *E*. *coli* cells were plated out on the opposite sides of lysogeny broth (LB)-agar plates containing ampicillin. After one day of growth at 37°C, three single colonies per plate were chosen for each candidate gene.

### Verification of the transformation

To verify the transformation, three single colonies from each transformation were selected and compared with the PCR product of the corresponding vector. Each PCR reaction was carried out in a 96-well PCR plate by adding 25 μL of a PCR master mix, containing the GoTaq® G2 DNA polymerase 0.25 units, colorless GoTaq® Flexi Buffer, MgCl_2_ 2.5 mM, dNTPs mix 0.4 mM (all products from Promega AG, Dübendorf, Switzerland), bovine serum albumin (BSA, Product B4287, Sigma-Aldrich, St. Louis, MO, USA), and 0.6 mg mL^-1^ (0.2 μM) forward (5’-GGTCGTCAGACTGTCGATGAAGCC-3’) and reverse primer (5’-TGTCCAACTCAGGAGAGCGTTCAC-3’) for each reaction. The sequences of the primers for the pMAL vector are provided by New England Biolabs (https://www.neb.com/en/faqs/2020/02/06/what-primers-should-i-use-to-sequence-the-ends-of-my-insert-after-i-clone-it-into-a-pmal-vector). Template DNA was added to the master mix (with 20 ng of the diluted plasmids as the control) or to the selected colonies of transformed cells. After being sealed, the PCR plate was freeze-thawed three times to break up the bacterial cells. The PCR program started at 95°C for 2 min, followed by 38 cycles of 94°C for 40 sec, 60°C for 40 sec, and 72°C for 2 min. A final elongation step was conducted at 72°C for 10 min. The PCR products were analyzed on a 2% agarose gel, and the sizes of the products were compared. Each vector had the same size as its transformed cell colonies (**S2 Fig).**

For storage of the bacterial cells, the colonies were transferred into 1 mL Eppendorf tubes containing 0.6 mL liquid LB-medium and 50 μg mL^-1^ ampicillin. Cells were grown at 37°C on a thermo-shaker (Thermomixer Comfort, Eppendorf SE, Hamburg, Germany) at 400 rpm overnight. After 0.6 mL of 20% of sterile glycerol was added, the cells were frozen in liquid nitrogen and stored at -80°C.

### Expression of the candidate genes

**Fig 2:**
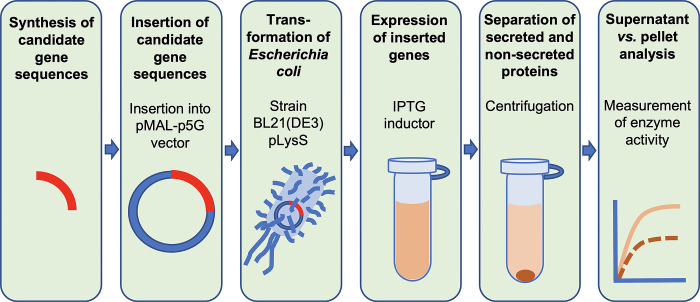
Workflow for functional validation of the proteins from candidate genes. For abbreviations see the Materials and Methods section.

Transformed *E*. *coli* strains from glycerol stocks were precultured overnight at 37°C in 10 mL Falcon tubes containing 3 mL of LB-medium and 50 μg mL^-1^ of ampicillin. Non-transformed control strains were grown in LB-broth without ampicillin. Then, 1 mL of each overnight culture was transferred to 10 mL LB-broth containing ampicillin. The cultures were grown at 37°C to an OD600 of 0.2–0.4 before induction. To induce gene expression, 40 μL isopropyl-β-D-1-thiogalactopyranosid IPTG (100 mM) was added (final concentration of IPTG: 0.4 mM). The bacteria with the induced genes were cultured overnight at 16°C, followed by centrifugation at 5,000 g for 30 min at 4°C. Pellets and supernatants were then stored at 4°C. An overview of the workflow for the functional validation of the proteins from candidate genes is shown in **Fig 2**.

### Esterase assay

The ability to degrade esters was tested with an esterase assay by applying p-nitrophenyl acetate (pNPA) [58,59]. The esterase assay is a proxy for the degradation of the plastic type polybutylene succinate (PBS) [58]. For this assay, 27.2 mg of pNPA (Product N8130, Sigma-Aldrich, St. Louis, USA) was dissolved in 1 mL of acetone, resulting in a 150 mM stock solution, and stored at -20°C. For the assay, the stock solution was diluted with 50 mM Tris-HCl (pH 8) and 0.1% Triton X-100 to reach a final pNPA concentration of 1 mM. To start the assay, 180 μL of this solution was mixed with 20 μL of supernatant in a 96-well plate (flat-bottom, transparent, polystyrene, Product 655162; Greiner Bio-One VACUETTE Schweiz GmbH, St. Gallen, Switzerland). The absorbance was measured at 405 nm every 2 min for 30 min (Tecan Infinite M200; Tecan Group AG, Männedorf, Switzerland). Esterase activity was expressed as the amount of the degradation product p-nitrophenol (pNP; Product 1048, Sigma-Aldrich, St. Louis, USA) resulting from the substrate pNPA and expressed in 1 μM pNP per min using a pNP standard curve [60].

### Plastic-degrading fluorescence assay

A fluorescence-based method adapted and applied by Rüthi et al. [24] was used to quantify hydrolysis of the plastic types BI-OPL and ecovio^®^. The approach is based on a fluorogenic probe embedded in the polymer matrix. Fluorescence is only detected once the fluorogenic probe is co-hydrolyzed together with the polymer.

To prepare 96-well microplates containing the polymer with the fluorogenic probe embedded in it, 300 mg of polymer was dissolved by sonication in a solvent mix containing 18 mL CHCl_3_ and 2 mL C_2_HCl_3_. A 20 mM stock solution of 4-methylumbelliferyl laurate (4-MUL; Santa Cruz Biotechnology Inc., Dallas, TX, USA) was prepared in C_2_HCl_3_. A 12.5 μL aliquot of 4-MUL stock solution was added to the dissolved polymer. A 100 μL aliquot of the resulting mixture was pipetted into each well of the 96-well plates (black, flat bottom, polypropylene, Product 655209; Greiner Bio-One VACUETTE Schweiz GmbH, St. Gallen, Switzerland), resulting in a total of 2.5 nM 4-MUL per well. The solvent was then evaporated by placing the plate onto refractory clay pre-warmed to 100°C, resulting in a polymer-coated 96-well plate.

After the transfer of 100 μL supernatant of the microbial culture to the 96-well plate, the samples were immediately measured using a plate reader (Tecan Infinite M200; Tecan Group AG, Männedorf, Switzerland). Excitation and emission wave lengths were set to 325 nm and 450 nm, respectively, and fluorescence was measured every 5 min for 12 h at 30°C and with a gain of 80.

Each plate contained a standard series to calculate the amount of hydrolyzed 4-MUL with a calibration curve. For this step, a 100 μM 4-methylumbelliferone (4-MU; Merck AG, Darmstadt, Germany) stock solution was prepared in methanol and diluted with Tris to 0 to 11 μM (in 1 μM steps) in the 96-well plates, reaching a final volume of 100 μL. The amount of hydrolyzed 4-MU was calculated for each sample using the calibration curve.

### Controls

Negative controls were the BL21(DE3)pLysS *E*. *coli* strains carrying the pMAL-p5G vector without any candidate gene or without the vector altogether. The positive control was the BL21(DE3)pLysS *E*. *coli* strain carrying the pMAL-p5G vector with a control gene for a lipase known to degrade PLA (PlaM4; National Center for Biotechnology Information NCBI: AB302136) [62]. However, the sequence was codon-optimized for *E*. *coli* and modified so that the amino acid sequence contained the pelB leader sequence and the His-tag, leading to a DNA sequence that differed from the sequence published by Mayumi et al. [61]. The gene sequence was cloned and expressed using the same procedure as for the candidate genes.

### Statistical analyses

The statistical significance of observed differences was assessed by applying factorial analysis of variance (ANOVA) with Fisher’s protected least significant difference, using StatView (v5.0, SAS Institute, Cary, NC, USA). Results from all statistical tests performed were considered significant at *P* < 0.05.

## Results

### Selection of candidate genes and characterization of their proteins

After screening the metagenomes, with a differential abundance analysis of the two metagenomes (plastisphere *vs*. bulk soil), we focused on 1,000 genes with the greatest log_2_-fold change. After a next step to select only those genes whose function was predicted to be correlated with plastic degradation, 214 sequences remained. Application of the SignalP server reduced the candidate group to 48 sequences. Further screening of these sequences to infer their function with greater confidence, with special attention to the identification of functional domains, resulted in a final group of nine candidate genes, A to I (**S1 File**). Out of the nine selected candidate genes, two had proteins assigned to hydrolases, two to glucosidases, one each to an esterase, a lipase, an acylase and a peptidase, and one remained uncharacterized (**Table 1**). The query cover varied greatly between 96% and 100%, while the identity was between 88.2% and 99.5%. The lengths of the predicted proteins were between 425 and 845 amino acids. The predicted bacterial genera to produce these proteins belonged mainly to *Caballeronia* and *Bradyrhizobium*. *Caballeronia* is a member of the Burkholderiales order and the Betaproteobacteria class, while *Bradyrhizobium* is a member of the Rhizobiales order and the Alphaproteobacteria class (**Table 1**). All taxonomic assignments were gram-negative bacteria.

**Table 1:** Best match of amino acid sequences (including signal peptides) from candidate genes A to I. Results are based on the National Center for Biotechnology Information *(*NCBI) blastp analysis.

	Predicted description	Query cover [%]	Identity [%]	Length [AA]^a^	Weight [kDa]	Predicted genus^b^	Log_2_-fold change^c^
**Protein A**	Uncharacterized protein	100	99.3	753	78.8	*Caballeronia*	11.8
**Protein B**	Patatin (lipase activity)	100	88.2	459	49.4	*Bradyrhizobium*	11.3
**Protein C**	β-Glucosidase	100	88.6	490	54.3	*Bradyrhizobium*	11.3
**Protein D**	Thioredoxin and six-hairpin glycosidase-like	100	90.0	598	66.6	*Bradyrhizobium*	11.5
**Protein E**	Serine hydrolase	96	94.3	453	49.9	*Caballeronia*	11.3
**Protein F**	SGNH/GDSL hydrolase^d^	100	99.5	425	45.2	*Caballeronia*	11.2
**Protein G**	Carboxylesterase	100	92.2	528	54.5	*Burkholderia*	11.3
**Protein H**	Penicillin acylase	100	97.8	845	90.5	*Variovorax*	11.1
**Protein I**	Peptidase	100	99.5	570	61.9	*Caballeronia*	11.2

^a^Length of the amino acid (AA) sequence.

^b^Predicted with Kaiju v1.7.4.

^c^Log_2_-fold change using DESeq2 for the differential gene expression analysis.

^d^Includes esterase and lipase. SGNH motif is a consensus amino acid sequence of Ser, Gly, Asn and His, where Gly and Asn donate proton to the oxyanion hole with Ser at the active site; GDSL motif is a consensus amino acid sequence of Gly, Asp, Ser and Leu around the active site Ser.

The use of more specific databases to describe the proteins from the candidate genes mostly yielded consistent results (**Table 2**). The protein produced from candidate gene B is most likely a phospholipase, from genes C and D a glucosidase, from gene E a β-lactamase, from gene G a carboxylesterase, from gene H a penicillin amidase, and from gene I a peptidase. Exceptions were gene A, for which most databases did not yield a hit, and gene F, which either corresponded to lipase, esterase or hydrolase (which also includes lipase and esterase) or yielded no hit (**Table 2**).

**Table 2:** Best match of amino acid sequences from candidate genes A to I using specific databases.

	eggNOG	Gene3D	Pfam	PMDB	SUPER-FAMILY	Foldseek
**Protein A**	NA^a^	NA	NA	Poly(3-hydroxy butyrate) depolymerase	Glycoside hydrolase	NA
**Protein B**	Patatin-like phospho- lipase	NA	Patatin-like phospho- lipase	NA	Acyltransferase/Acylhydrolase	Ca-independent phosphor lipase
**Protein C**	Glycoside hydrolase	NA	Glycoside hydrolase	NA	Glycoside hydrolase	β-Glycosidase
**Protein D**	Thiore-doxin-like	Six-hairpin glycosidase	Glycosyl hydrolase	NA	Thioredoxin-like	Thioredoxin-like
**Protein E**	Carboxy- peptidase	NA	β-Lactamase	Carboxyl- esterase	β- Lactamase	β-Lactamase
**Protein F**	Lipase	Hydrolase	Hydrolase- type esterase	NA	NA	GDSL-like lipase/acyl- hydrolase^b^
**Protein G**	Carboxyl- esterase	α/β-Hydrolase	Carboxyl- esterase	Carboxylic ester hydrolase	α/β- Hydrolase	Carboxylic ester hydrolase
**Protein H**	Penicillin amidase	Penicillin amidase	Penicillin amidase	NA	Nucleophile amino- hydrolase	Acylase
**Protein I**	Peptidase	α/β- Hydrolase	Peptidase	NA	α/β- Hydrolase	Carboxy- peptidase

^a^NA: Not applicable or no hit.

^b^GDSL: GDSL motif is a consensus amino acid sequence of Gly, Asp, Ser and Leu around the active site Ser.

### Prediction of structural elements of the proteins

Using the SignalP server, it was found that three of the nine proteins produced from the nine candidate genes had the standard secretory signal peptides transported by the Sec translocon and and cleaved by signal peptidase I, three had the lipoprotein signal peptides transported by the Sec translocon and and cleaved by signal peptidase II, and three had the signal peptides transported by the Tat translocon and cleaved by signal peptidase I (**Table 3**). The cleavage sites of the peptides for all proteins of the candidate genes were between the 16th and 50th amino acid. The probabilities of the cleavage site position were 80%–99%. The screening for transmembrane domains revealed that all proteins of the candidate genes lacked such domains (**Table 3**).

**Table 3:** Prediction of the structural elements of proteins from candidate genes. Predicted signal peptide properties are from the SignalP v5.0 server, predicted transmembrane domains (TMD) are from the TMHMM v2.0 server, pocket properties are from the P2Rank software, and the similarity evaluations are from the Plastics Microbial Biodegradation Database (PMBD).

	Signal peptide typo-logy	Signal peptide cleavage site	Signal peptide proba-bility	TMD^a^	Pocket score	Pocket proba-bility	Pocket AA^b^	Pocket conser-vation	Simila-rity eva-luation
**Protein A**	Sec/SPI^c^	25-26/ AQA-QS	0.94	0	9.5	0.55	19	-	0.57 PHB^f^ degrader
**Protein B**	Sec/SPII^d^	16-17/ LLG-CA	0.99	0	8.3	0.49	25	1.20	0.95 Pht.^g^ degrader
**Protein C**	Tat/SPI^e^	28-29/ AKA-AD	0.91	0	12.0	0.66	14	0.41	No relevant degrader
**Protein D**	Tat/SPI	20-21/ SFA-DP	0.99	0	11.5	0.64	30	0.36	0.99 PVA^h^ degrader
**Protein E**	Tat/SPI	49-50/ AEA-AE	0.87	0	20.7	0.85	23	0.83	0.89 PHA^i^ degrader
**Protein F**	Sec/SPI	26-27/ ALA-DD	0.97	0	10.2	0.59	18	1.92	0.89 PHA degrader
**Protein G**	Sec/SPII	23-24/ VAS-CG	0.99	0	20.1	0.84	29	0.91	0.97 PVA degrader
**Protein H**	Sec/SPII	22-23/ LTA-CG	0.99	0	6.8	0.38	11	1.58	0.99 PHB degrader
**Protein I**	Sec/SPI	22-23/ VMA-QE	0.80	0	33.0	0.93	33	1.77	0.99 PHB degrader

^a^TMD: Number of transmembrane domains.

^b^AA: Number of amino acids involved in the pocket.

^c^Sec/SPI: Standard secretory signal peptides transported by the Sec translocon and cleaved by signal peptidase I.

^d^Sec/SPII: Lipoprotein signal peptides transported by the Sec translocon and cleaved by signal/peptidase II.

^e^Tat/SPI: Tat signal peptides transported by the Tat translocon and cleaved by signal peptidase I.

^f^PHB: Polyhydroxybutyrate.

^g^Pht.: Phthalate.

^h^PVA: Polyvinyl acetate.

^i^PHA: Polyhydroxyalkanoate.

Furthermore, the protein sequences were screened for active pockets, as this is a prerequisite for hydrolysis. Pockets in proteins are cavities on the surface or in the interior of a protein that possess suitable properties for binding a ligand. The scores of the main pockets, which represent the raw scores of the residues belonging to the predicted pockets, had values between 6.8 and 20.7. The probability scores were between 0.38 and 0.93, and the counts of amino acids involved in the predicted pockets were between 11 and 33. The pocket conservation scores, representing the average conservation scores calculated for all the residues belonging to the predicted pocket, were between 0.41 and 1.92 (**Table 3**).

Similarity-based screening of the potential plastic-degrading capability of proteins from candidate genes indicated similarities between sequences and enzymes related to plastic biodegradation for the groups PVA, PHB, PHA or phthalate. The similarities for all proteins of the candidate genes were 0.89 or greater, except for the protein of candidate gene A, which had a value of only 0.57, and the protein of candidate gene C, which had no similarity at all (**Table 3**).

### Plastic-degrading activities of proteins from candidate genes

Nucleotide sequences of the nine candidate genes (**S2 File**) were synthesized and cloned into a compatible *E*. *coli* strain. Colony PCR and electrophoretic analysis showed that the transformation procedures were successful and contained the correct inserts (**S3 Fig**). Enzymatic activities of the produced proteins of the candidate genes A to I were verified using either an esterase assay or a plastic degradation assay. The comparison between the supernatant and the pellet showed that the activity in the supernatant in the esterase assay was about twice as high as in the pellet (**S4 Fig**). Consequently, only the supernatant was used for all further assays. Of the proteins of the nine candidate genes, five had significantly increased activities, and one, the protein from candidate gene F, was clearly the most active (**Table 4**). While the proteins of candidate genes B, D, G and I showed significantly increased formation of pNP compared with the negative controls X and Y without inserted genes, by about 0.5 μM pNP min^-1^, the protein of gene F showed significantly increased formation of pNP, by about 8.5 μM pNP min^-1^, meaning that the total amount of pNP was more than doubled. Interestingly, the positive control Z, with a protein having degrading capabilities for PLA, was also significantly enhanced, by about 1.9 μM pNP min^-1^ (**Table 4**).

**Table 4:** Enzymatic activities of proteins from candidate genes A to I and of controls X to Z determined by applying esterase and plastic degradation fluorescence assays. Values are means ± SE (n = 3). Different letters indicate significant differences (*P* <0.05) using one-way analysis of variance (ANOVA) followed by a least significant difference (LSD) test. Values (and the corresponding letters) that are significantly different from the X-control are in bold.

	Esterase assay [μM pNP min^-1^]	Fluorescence assay BI-OPL [μM 4-MU h^-1^]	Fluorescence assay ecovio^®^ [μM 4-MU h^-1^]
**Protein A**	6.01 ± 0.16 10^−3^ d	NM^a^	NM
**Protein B**	**6.84** ± 0.15 **10**^**−3**^ **c**	-1.38 ± 0.12 10^−2^ b	-1.17 ± 0.08 10^−2^ b
**Protein C**	6.01 ± 0.11 10^−3^ d	NM	NM
**Protein D**	**6.88** ± 0.08 **10**^**−3**^ **c**	-1.19 ± 0.07 10^−2^ b	-1.10 ± 0.04 10^−2^ b
**Protein E**	6.15 ± 0.09 10^−3^ d	NM	NM
**Protein F**	**14.72** ± 0.09 **10**^**−3**^ **a**	-0.94 ± 0.11 10^−2^ b	-0.60 ± 0.10 10^−2^ b
**Protein G**	**6.58** ± 0.10 **10**^**−3**^ **c**	-1.51 ± 0.06 10^−2^ b	-1.28 ± 0.08 10^−2^ b
**Protein H**	6.17 ± 0.08 10^−3^ d	NM	NM
**Protein I**	**6.57** ± 0.06 **10**^**−3**^ **c**	-1.46 ± 0.06 10^−2^ b	-1.03 ± 0.05 10^−2^ b
**Control X** ^**b**^	6.13 ± 0.06 10^−3^ d	-1.39 ± 0.05 10^−2^ b	-0.78 ± 0.07 10^−2^ b
**Control Y** ^**c**^	6.00 ± 0.07 10^−3^ d	-1.95 ± 0.15 10^−2^ b	-0.72 ± 0.09 10^−2^ b
**Control Z** ^**d**^	**7.95 ± 0.19 10**^**−3**^ b	**22.89 ± 0.65 10**^**−2**^ **a**	**23.28 ± 0.65 10**^**−2**^ **a**

^a^NM: Not measured.

^b^X: Negative control 1: *Escherichia coli* with empty pMAL-p5G vector.

^c^Y: Negative control 2: *Escherichia coli* without vector and without a gene insertion.

^d^Z: Positive control: *Escherichia coli* with pMAL-p5G vector and with a gene that produces a PLA-degrading protein (modified PlaM4 [61]).

The fluorescence assays for plastic degradation of BI-OPL and ecovio^®^ were only carried out with the proteins of the candidate genes B, D, F, G and I, which showed significant activity in the esterase assay. In the fluorescence assays, all proteins tested showed no significant activity, with values below 0 μM 4-MU h^-1^. The negative controls X and Y also showed no significant activity. In contrast, the positive control Z, which contains a PLA-degrading gene insert, showed a very high activity with values around 23 μM 4-MU h^-1^ (**Table 4**).

## Discussion

### Metagenomic mining of the plastisphere

The plastisphere is believed to selectively attract microorganisms that are capable of degrading and utilizing this carbon source for growth [20]. Therefore, exploration of the plastisphere using metagenomic methods to find microorganisms or genes involved in plastic degradation is a targeted approach with great potential [62–64]. In pioneering studies, this approach was applied to the plastisphere of Arctic and alpine cryosphere soils to decipher little-known or unknown microorganisms or enzymes (e.g. [24,25]). In these studies, the fungal genera *Lachnellula* and *Neodevrisia* in particular were found to be attracted to the potentially degradable plastics BI-OPL and ecovio^®^, both containing PBAT and PLA. In contrast, PE plastic did not attract any particular microorganisms, as the microbial community of the plastisphere soil did not differ from that of the surrounding bulk soil. The metagenomic analyses showed that mainly α/β-hydrolases were involved in the degradation of the BI-OPL and ecovio^®^ plastisphere.

*In silico* metagenomic mining approaches of the plastisphere, however, are still rare. Mayumi et al. [61] constructed a metagenomic library consisting of the DNA extracted from PLA disks buried in compost. They identified three PLA-degrading genes encoding lipase or hydrolase. The most active one, a homolog of a lipase of *Bacillus*, was used in the present study as a positive control.

Our approach using the *in silico* method led to a small group of candidate genes encoding mainly lipases or esterases. We carefully applied analytical and biochemical parameters in the selection of genes of interest to avoid false positives, and we specifically considered the fact that enzymes are secreted. When predicting enzymes from databases, there is a potential risk of including false positives, particularly through the unintentional but unfiltered use of incorrectly annotated GenBank entries and/or the failure to carefully check the obtained references using rigorous analytical and biochemical parameters [5].

We narrowed the potential genes through several procedures and steps. After applying differential abundance analyses with DEseq2, we focused on 1,000 genes with the greatest log_2_-fold change within the initial metagenomic output. Screening for signal peptides and transmembrane domains was performed as an additional step, as the secretion of proteins is a relevant criterion and the presence of domains can cause difficulties due to their hydrophobicity and potential toxicity to the host cell [65]. In our case, only proteins lacking transmembrane domains belonged to the candidate group. In addition, the TMHMM server outputs the expected number of amino acids involved and the overall probability that the N-terminus of the protein is on the cytoplasmic side of the membrane [66].

Since we assumed that plastic-degrading enzymes have a region responsible for interacting with the substrate, we uploaded the 3D structures into the P2Rank software to check for the presence of interaction pockets. P2Rank achieves high prediction success rates by scoring and clustering points on the solvent-accessible surface of the protein. The ligand stability score of each point is determined by a machine-learning-based model trained on the dataset of known protein-ligand complexes [51]. As output, the software provides the probability of the presence of pockets together with other parameters concerning the reliability of the prediction. Among these parameters, the conservation score is the one that captures the extent of variation in each amino acid residue, with values from 0 (representing non-conserved residues) to 1 (representing highly conserved residues). The closer the value is to 1, the more reliable the prediction made by the software, which is based directly on multiple sequence alignment with already known protein structures [52].

The potential ability to degrade plastics was further investigated using PMBD, a database for microbial plastic biodegradation, which enables analysis of the similarity between a user-supplied sequence and the sequences present in the database using a hidden Markov model alignment tool (HMMER) [55]. As the name suggests, PMBD is a collection of 79 literature-confirmed plastic-degrading enzymes and more than 8,000 automatically annotated enzyme sequences thought to be involved in the biodegradation of plastics. This database provides the user with a tool called “function prediction”, implemented by a convolutional neural network (CNN) model, which predicts the function of the uploaded protein for plastic biodegradation, based on a similarity score between its sequence and an enzyme related to plastic [55]. Six of our nine candidate genes were found to be related to PHA, PHB, or PVA degraders, with a probability of at least 0.89, suggesting that the selected candidate genes have a high potential to degrade plastics.

### Functional evaluation

The function-based assay for the determination of active enzymes using an esterase assay showed more than double the enzymatic activity for the protein from candidate gene F than for the negative controls. The proteins from candidate genes B, D, G and I had only a slight, but still significant, increase in esterase activity compared with the negative controls. However, the proteins from all our candidate genes, as well as the negative controls, lacked any activity in the fluorescence assay representing BI-OPL or ecovio^®^ plastic degradation. The positive control containing the PLA-degrading gene PlaM4 (published by Mayumi et al. [61]), in contrast, showed more than 20 times higher degradation activity for both BI-OPL and ecovio^®^ compared with the negative controls. Mayumi et al. [61] observed that the purified PLA depolymerase PlaM4, in addition to PLA, has also the capability to degrade PBS, PBSA, PES, PCL and PHB. They found the sequence be homologous to a *Bacillus* lipase.

**Fig 3:**
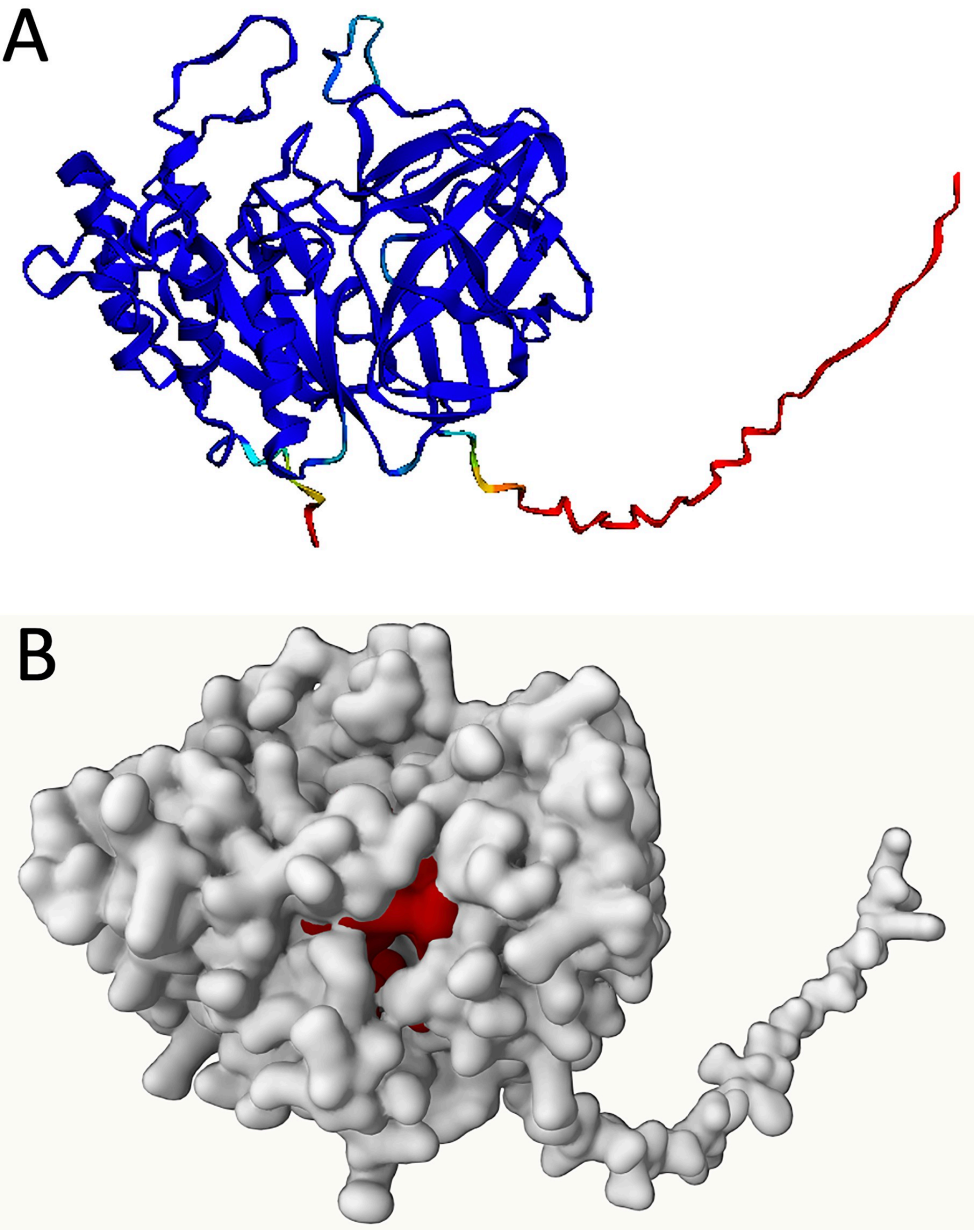
Model predictions for the protein from candidate gene F (hydrolase-type esterase). **(A)** AlphaFold2 prediction of the protein structure. Blue corresponds to very high (>90%), light blue to high (80%), green to moderate (70%), yellow to low (60%), and red to very low (<50%) confidence. **(B)** P2Rank prediction for the AlphaFold2 model. The main pocket on the structure is shown in red.

The protein from candidate gene F, found to be the most suitable protein in our study, belongs to the GDSL-like lipase/acylhydrolase family, with Pfam characterizing the enzyme having a SGNH hydrolase-type esterase domain. SignalP proposed a signal peptide of 26 amino acids, with the standard secretory type Sec/SPI. Among all candidate genes, P2Rank assigned candidate gene F a relatively low pocket score (10.2) and low probability score (0.59), but with a high value in conservation score (1.92) (**Table 3** and **Fig 3**). With a similarity score of 0.89, the protein from candidate gene F belongs to the known PHA degraders, with *Caballeronia* being the most likely bacterial genus. The *Caballeronia* genus belongs to the Burkholderiaceae family, whose members are able to fix nitrogen and promote plant growth. Some members of *Caballeronia* have been found in acidic soils and in the mycorrhizosphere of trees [67,68]. In general, members of Burkholderiaceae are known to be capable of degrading a vast array of aromatic compounds, as described in the review by Pérez-Pantoja et al. [69], including several pollutants (e.g. [70]).

Overall, the fluorescence assays used in this study could be powerful tools for assessing plastic degradation because real plastic compounds are involved in the test, and not just a molecule as in the esterase assay. Our tests proved to be an effective tool, as the positive control gene Z, the PLA depolymerase PlaM4, was able to degrade both BI-OPL and ecovio^®^ at a high rate. For that depolymerase, SignalP indicated a signal peptide of 27 amino acids, corresponding to the standard Sec/SPI secretion type, and P2Rank indicated a pocket score of 13.9, a probability score of 0.72, a count of amino acids of 23, and a pocket conservation score of 1.66 (**S5 Fig**). The 3D structure of that polymerase is stored in a public database (https://alphafold.ebi.ac.uk/entry/A4UZ10). Our approach was successful when we applied the esterase assay, but in the future more candidate genes should be screened. An alternative approach would be to isolate candidate genes directly from the metagenome of organisms induced by the plastisphere, e.g. members of the fungi *Neodevrisia* and *Lachnellula* or of the actinobacteria *Umezawaea* and *Streptomyces* [24].

### Genomic potential of microbial plastic degradation

Genome mining has become a promising tool for the discovery of new plastic-degrading enzymes in recent years [5,64,71]. In particular, the heterologous expression-based approach for finding new plastic-degrading enzymes offers distinct advantages First, it bypasses the cultivation step, which is often challenging for cold-adapted microorganisms. Second, virtually any gene of interest available in public repositories can be easily synthesized and used directly for heterologous expression without the need for the original organism. Third, knowing the enzyme-coding sequence opens up additional avenues of investigation, such as conducting sequence-based modeling to predict enzyme activity and properties *in silico* prior to testing, or conducting genetic engineering experiments to improve or adapt the enzyme [16,72]. In addition, since genes are often silent in organisms, meaning that they are not expressed under laboratory conditions, the heterologous expression-based approach can solve this problem, or gene expression can be induced and enhanced by heterologous expression, resulting in much higher enzyme yields [73,74]. Metagenomic mining approaches to search for plastic-degrading enzymes have already yielded promising results, as Popovic et al. [75] and Hajighasemi et al. [76] used a set of environmental metagenomes to find new carboxylesterase families including enzymes with PLA- and polycaprolactone-degrading activity. Moreover, by screening metagenomes from compost piles, Sonnendecker et al. [18] found a highly active polyester hydrolase that completely hydrolyzes amorphous PET films into terephthalic acid, and Qi et al. [77] discovered new PET hydrolases in a glacial metagenome with low sequence identity to known active PET hydrolases, possibly representing a new PET hydrolase class.

As current cultivation technologies have not yet led to the identification of highly active enzymes for most plastics, the diversity of uncultured microorganisms and the so-called dark-matter proteins offer a promising source for the identification of plastic-degrading enzymes [78]. In addition, the further development of smart search algorithms for the evaluation of metagenome datasets is an important part of the research [5]. Such algorithms include the well-designed and experimentally verified hidden Markov models (HMM), which showed promising results in a recent study [79]. Related to the search for active plastic-degrading enzymes is the use of well-curated databases specialized in plastic degradation, e.g. PMBD [55], PlasticDB [80], and PAZy [9]. All such databases provide an up-to-date overview of potential and verified enzymes acting on plastics, but with individual specifications, e.g. PAZy contains only functionally verified and manually curated enzymes, while PMBD and PlasticDB contain larger datasets of predicted enzymes and microorganisms detected in enrichment cultures [5]. Gambarini et al. [81] found a total of 16,170 putative orthologues for plastic degradation in 6,000 microbial species belonging to 12 phyla.

In the future, structure-based searches will be improved through structure-predicting tools such as AlphaFold2 and Robetta [5]. In our study, we obtained detailed information on the pocket properties by applying AlphaFold2 followed by P2Rank software. In addition, *in vitro* transcription and translation technologies, in combination with HMM-based screening, will be able to deliver data within a relatively short time, making it possible to circumvent time-consuming protein production in heterologous hosts [82].

We applied a fluorescence-based assay using two different plastic types. This sensitive method was developed by Zumstein et al. [83] and modified and applied by Cerri [84] and Rüthi et al. [24] for the first time. In this method, a fluorescent compound is bound to the plastic material, and when an enzyme breaks down this bond, a fluorescent signal is emitted. This method therefore directly measures the degradation of plastics and is very sensitive due to the fluorescence. This method could be extended to other types of plastic, including those previously known to be difficult for microorganisms to degrade, such as PE and PP.

In the future, one focus in the search for plastic-degrading enzymes should be the plastic recycling process, e.g. PET degradation into its molecular components, to deliver the basis to reconstruct PET [18,85]. An additional research direction should be the search for enzymes that can degrade hardly degradable plastic types, such as PE, PP and PVC [5]. Our shotgun DNA metagenomics approach does not allow us to predict whether the differentially abundant genes we selected would be expressed simultaneously. However, we are convinced that the identified genes with a log_2_-fold change of 11 are relevant for plastic biodegradation. In addition to metagenomics, metatranscriptomics would be a useful method, as it indicates the expression of enzymatic machinery capable of plastic degradation. By applying metatranscriptomics to PE films, McLean et al. [11] found a variety of plastic-associated genes (e.g. PETase), indicating the presence of potential enzymes for plastic degradation. Besides a metagenomic approach, a proteomic approach could be a powerful method in the future [86,87]. Recently, Messer et al. [88] discovered new enzymes in the plastisphere using a multi-omics and a comparative metaproteomic approach. With this targeted search strategy, they identified plastic biodegradation enzymes on thin biofilms, such as polyamidase, hydrolase and depolymerase. The authors concluded that their method helps to clarify the functioning of plastispheres and offers new perspectives for bioengineering and a better assessment of the risks of plastic pollution.

## Conclusion

Here, we showed that the application of *in silico* screening of a metagenome followed by functional validation screening has a high potential to find new enzymes capable of plastic degradation. Our approach led to the discovery of a new hydrolase-type esterase with high esterase activity from an uncultured bacterium predicted to be *Caballeronia*. The esterase assay is a proxy for the degradation of the plastic type polybutylene succinate (PBS), yet a comparison with the PMBD revealed that *Caballeronia* is not listed. Instead, esterases are only known from *Thermobifida*, and a similarity analysis yielded a similarity score of only 0.89 with all known plastic-degrading enzymes. Although no suitable plastic-degrading enzyme was found in the plastisphere of our experiment to degrade BI-OPL or ecovio^®^, the PLA depolymerase used as a positive control was found to degrade these two plastic types with high efficiency. Both plastics contain PBAT and PLA. Therefore, combining new and specialized databases in combination with sensitive and easy-to-use plastic degradation assays for function validation seems to be a powerful tool in the search for new plastic-degrading enzymes. Our results suggest that it is possible to screen soils from regions that are genetically poorly understood, such as cold alpine and Arctic zones, using these metagenomic methods, and thus to assess their biotechnological potential.

## Supporting information

S1 Fig The pMAl-p5G vector from GeneScript® (https://www.genscript.com/location.php?href=/gsfiles/vector-map/bacteria/pMAl-p5-g.pdf). **(A)** Vector map and **(B)** sequence of the most relevant parts of the vector. For a detailed description see https://www.snapgene.com/plasmids/basic_cloning_vectors/pMAL-p5G. AmpR: ampicillin resistance, Lac I: lactose repressor, MBP: maltose-binding protein, MCS: multiple cloning site, Ori: origin of replication.
(TIF)

S2 Fig Evaluation of the transformation procedure using a 2% agarose gel. For each of the candidate genes B, C, D, E and F, the first column is loaded with the pure pMAL-p5G vector (V), and the following three columns are loaded with three different transformed *Escherichia coli* colonies containing the pMAL-p5G vector (1, 2 and 3). The presence of bands in the column of the pure vector demonstrates that the primers exploited were appropriate, while the presence of bands in the other three columns are proof that the transformation procedure was successful.
(TIF)

S3 Fig SDS-PAGE of proteins from transformed *Escherichia coli* strains containing candidate genes A, B, C, F and H before and after induction of expression with IPTG (isopropyl ß-D-1-thiogalactopyranoside). Red arrows indicate the proteins of the candidate genes bound to the maltose-binding protein (43 kDa): A: 122 kDa, B: 92 kDa, C: 97 kDa, F: 88 kDa, H: 134 kDa.
(TIF)

S4 Fig Time course in minutes of enzymatic cleavage of p-nitrophenol (pNP) from p-nitrophenyl acetate (pNPA) by the protein of candidate gene F from supernatant and from pellet in the esterase assay. (TIF)

S5 Fig P2Rank prediction for the positive control protein Z (PLA depolymerase PlaM4; Mayumi et al. 2008). The main pocket on the structure is shown in red. Pocket score: 13.9, probability score: 0.72, amino acids count: 23, pocket conservation score: 1.66.
(TIF)

S1 File Amino acid sequences of the proteins from candidate genes A to I and from the positive control gene Z. The signal peptides are indicated in red letters.
(PDF)

S2 File *Escherichia coli* codon optimized nucleotide sequences of candidate genes A to I and the positive control gene Z used for cloning. (PDF)

S1 Raw images (PDF)
