# Supplementary figures and images for: Searching for new plastic-degrading enzymes from the plastisphere of alpine soils using a metagenomic mining approach
Source: PLoS One. 2024 Apr 5;19(4):e0300503. doi: 10.1371/journal.pone.0300503 (PMC10997104; doi:10.1371/journal.pone.0300503)

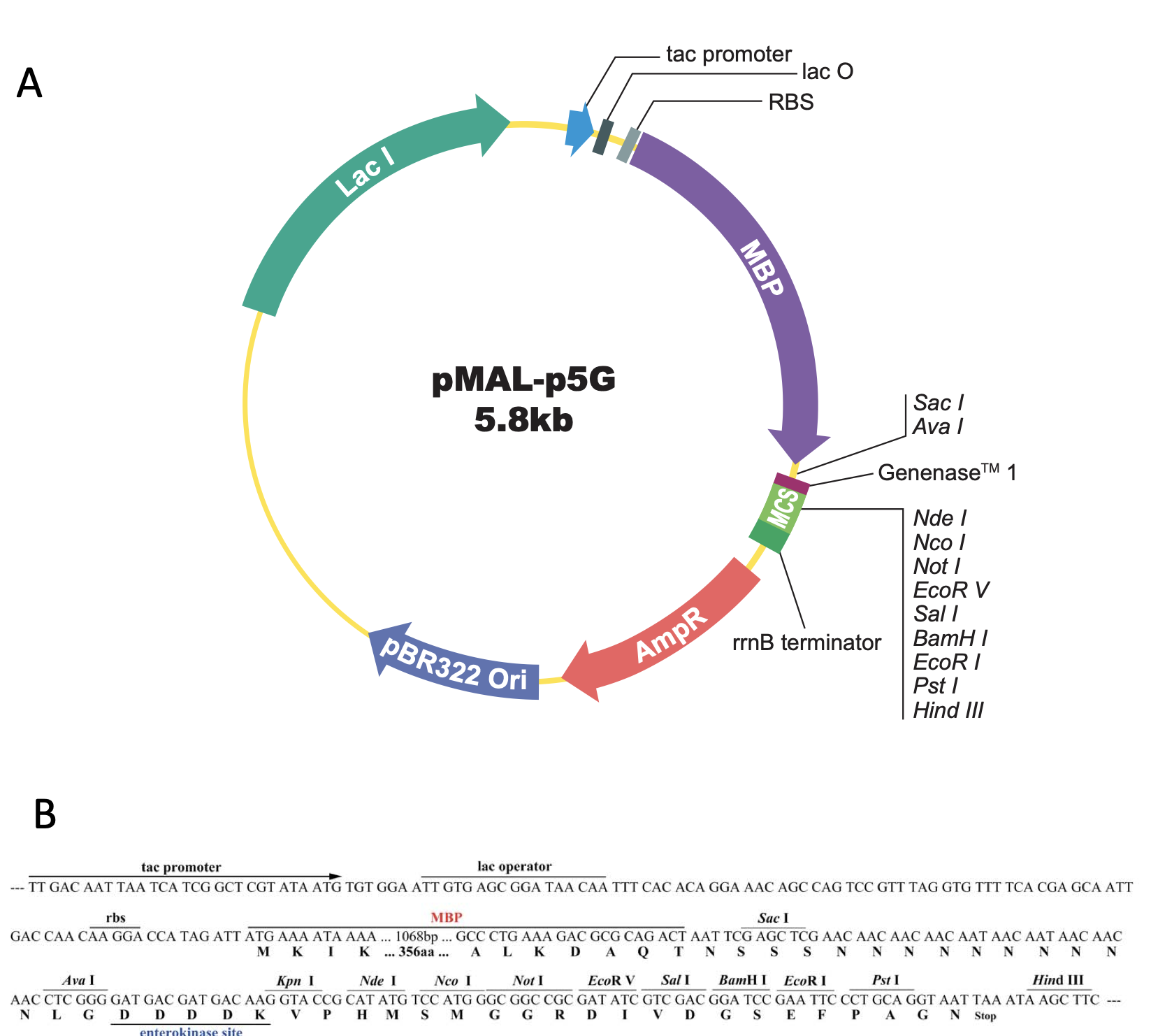

Supplement: S1 Fig — (A) Vector map and (B) sequence of the most relevant parts of the vector. For a detailed description see https://www.snapgene.com/plasmids/basic_cloning_vectors/pMAL-p5G. AmpR: ampicillin resistance, Lac I: lactose repressor, MBP: maltose-binding protein, MCS: multiple cloning site, Ori: origin of replication. (TIF) [file pone.0300503.s001.tif]

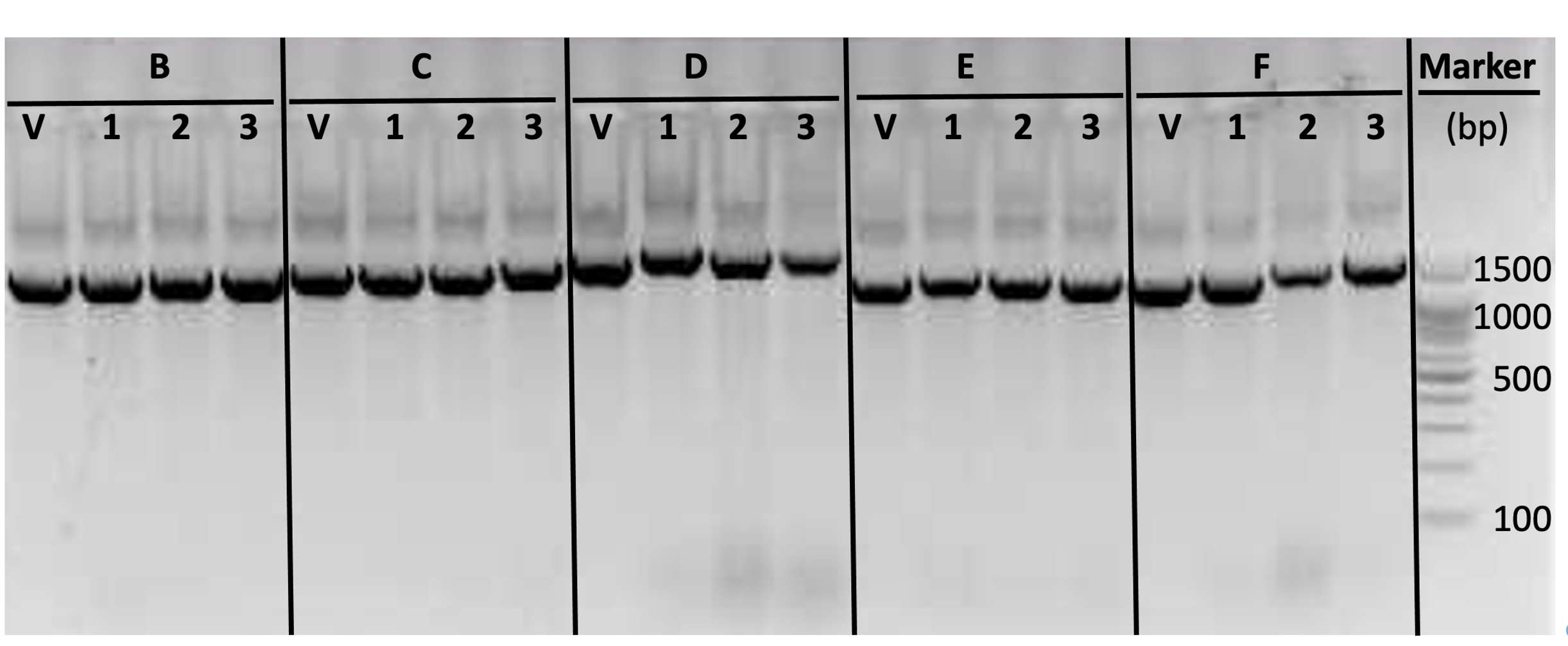

Supplement: S2 Fig — For each of the candidate genes B, C, D, E and F, the first column is loaded with the pure pMAL-p5G vector (V), and the following three columns are loaded with three different transformed Escherichia coli colonies containing the pMAL-p5G vector (1, 2 and 3). The presence of bands in the column of the pure vector demonstrates that the primers exploited were appropriate, while the presence of bands in the other three columns are proof that the transformation procedure was successful. (TIF) [file pone.0300503.s002.tif]

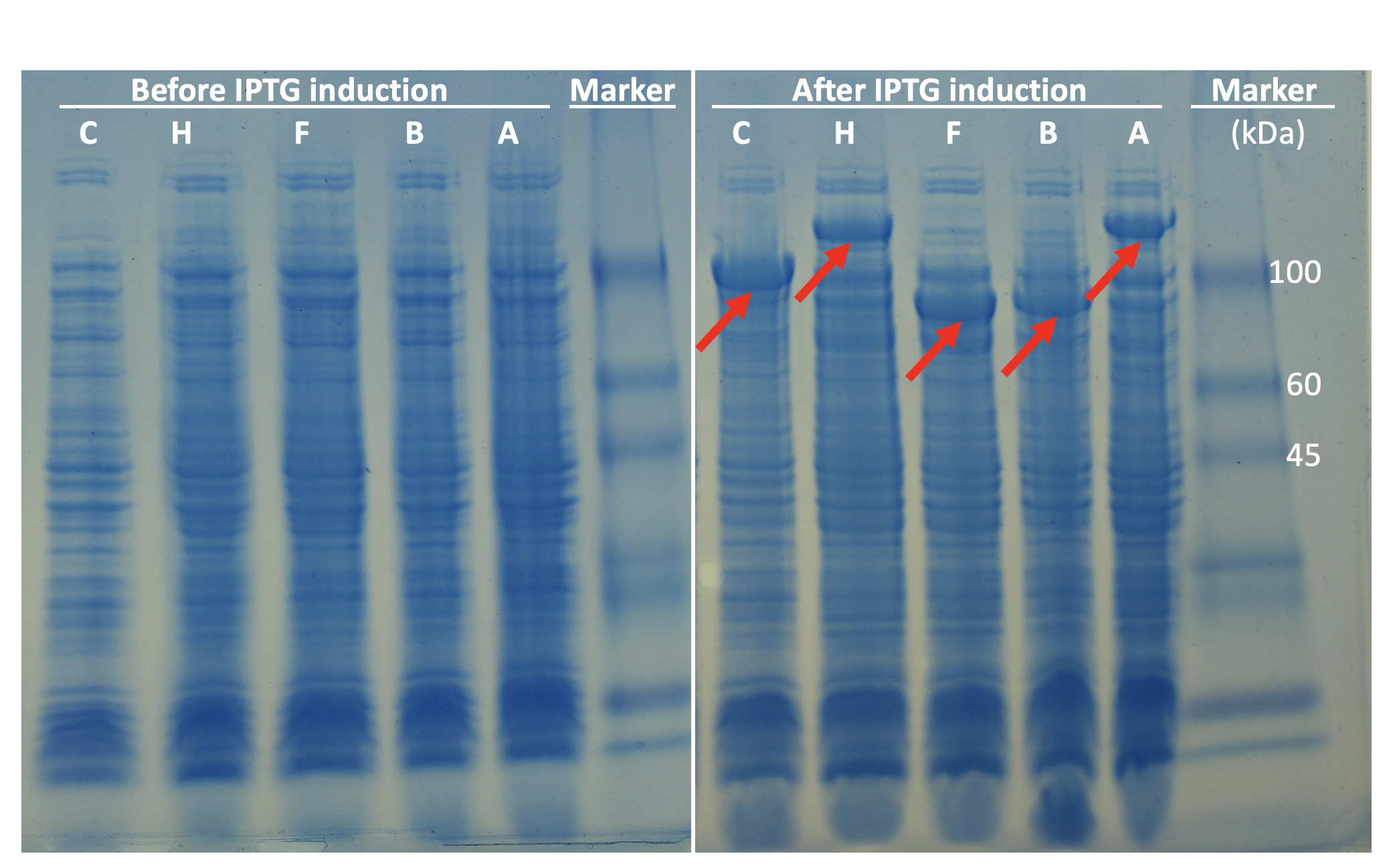

Supplement: S3 Fig — Red arrows indicate the proteins of the candidate genes bound to the maltose-binding protein (43 kDa): A: 122 kDa, B: 92 kDa, C: 97 kDa, F: 88 kDa, H: 134 kDa. (TIF) [file pone.0300503.s003.tif]

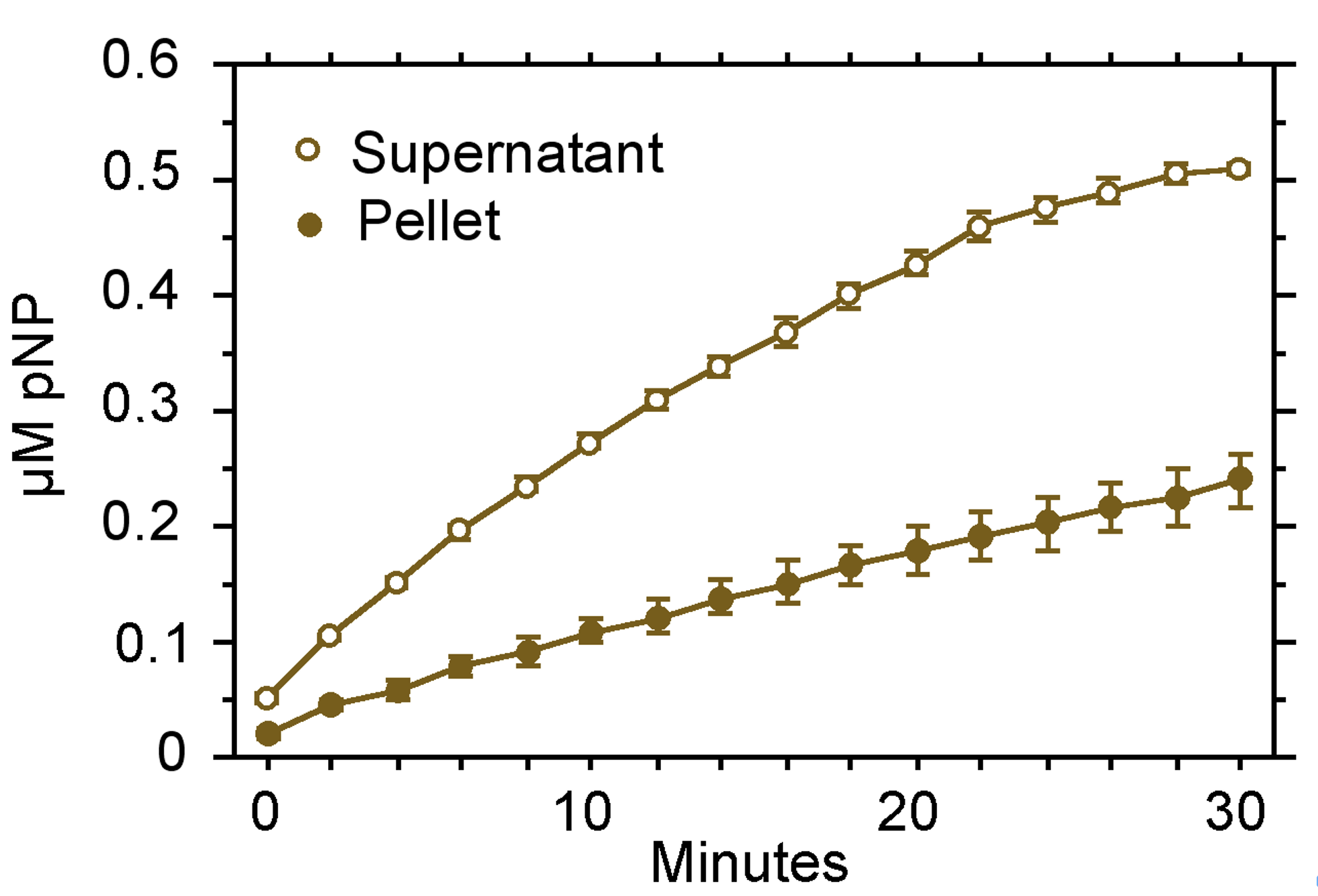

Supplement: S4 Fig — (TIF) [file pone.0300503.s004.tif]

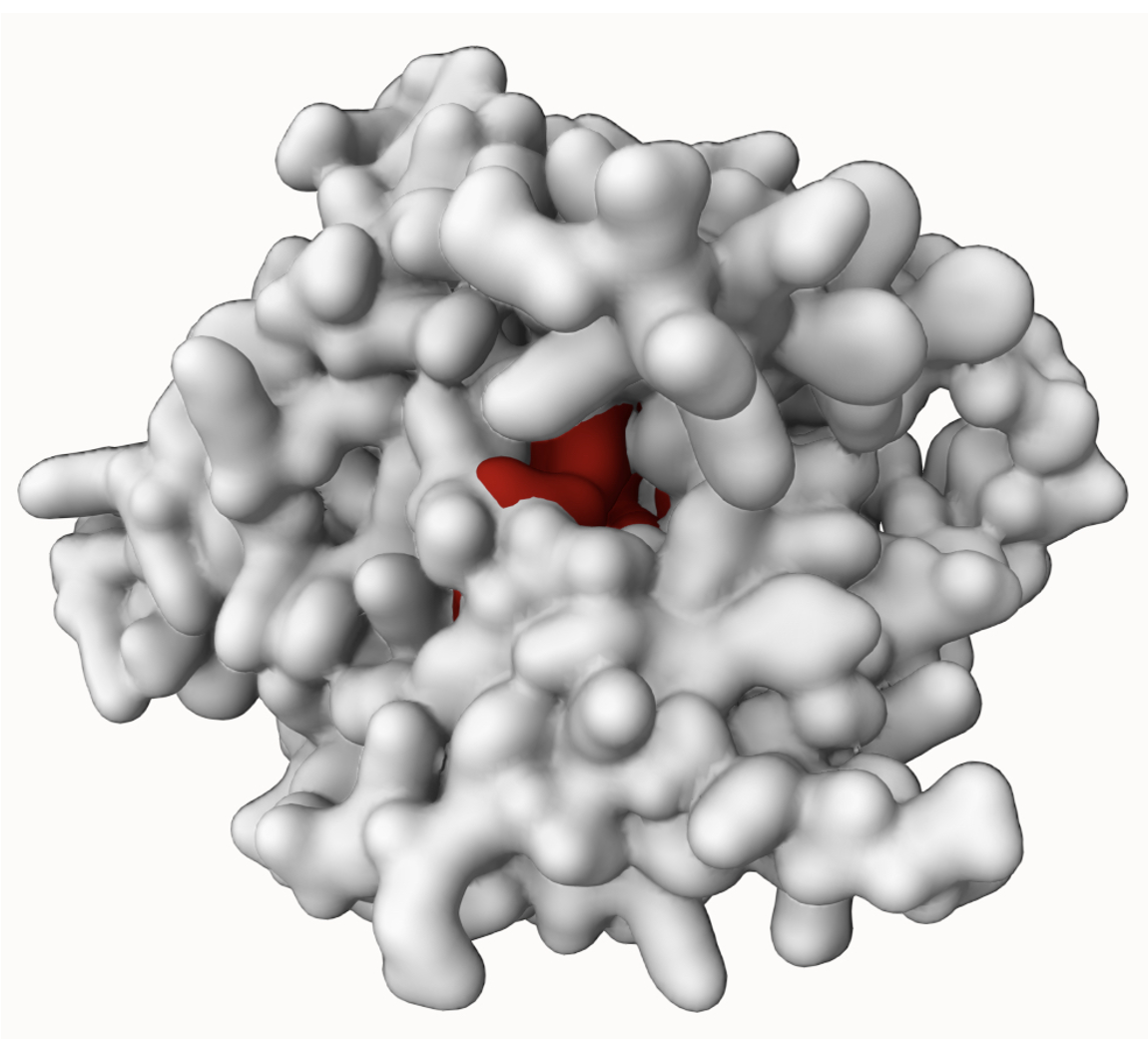

Supplement: S5 Fig — The main pocket on the structure is shown in red. Pocket score: 13.9, probability score: 0.72, amino acids count: 23, pocket conservation score: 1.66. (TIF) [file pone.0300503.s005.tif]

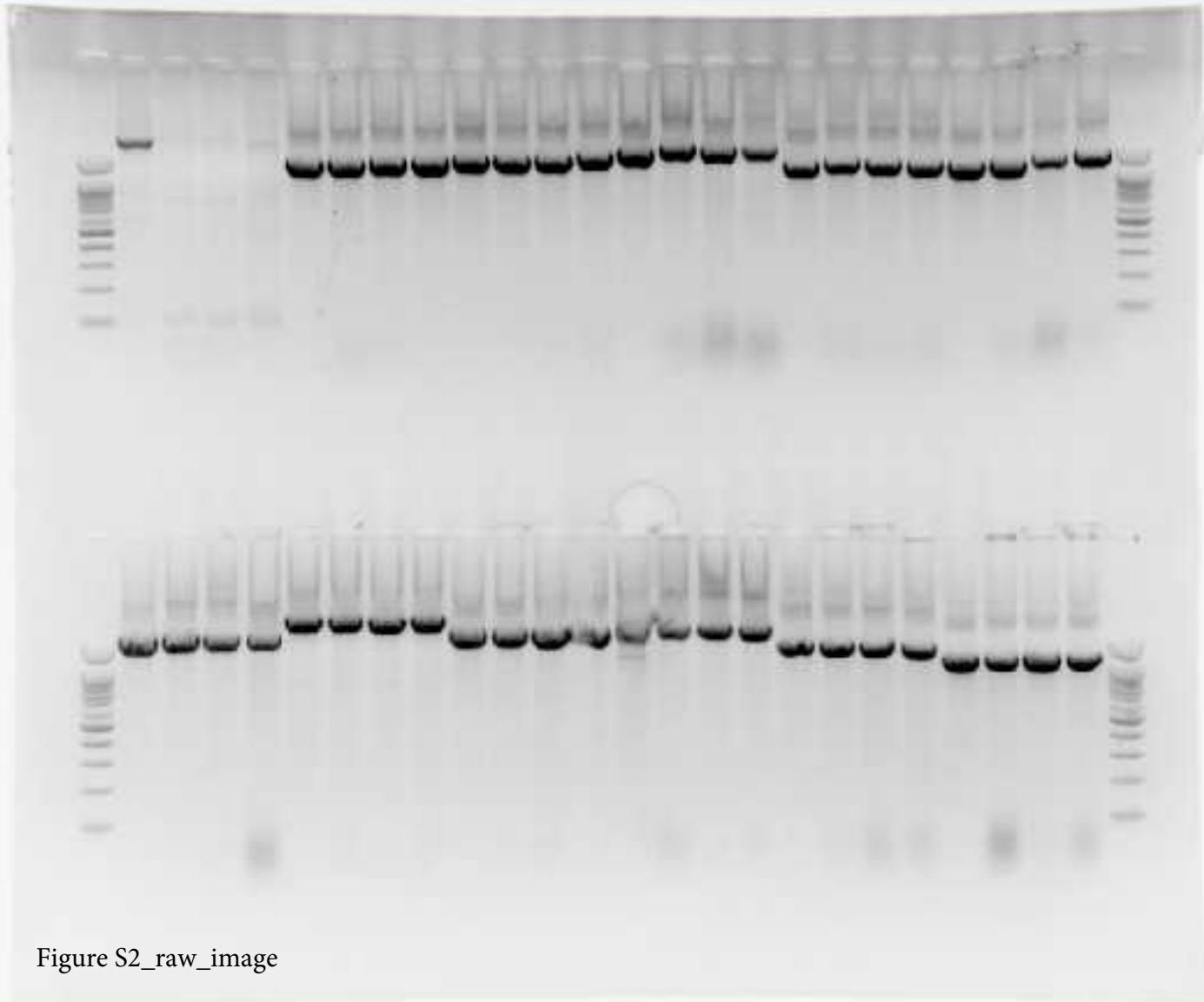

Figure S2\_raw\_image

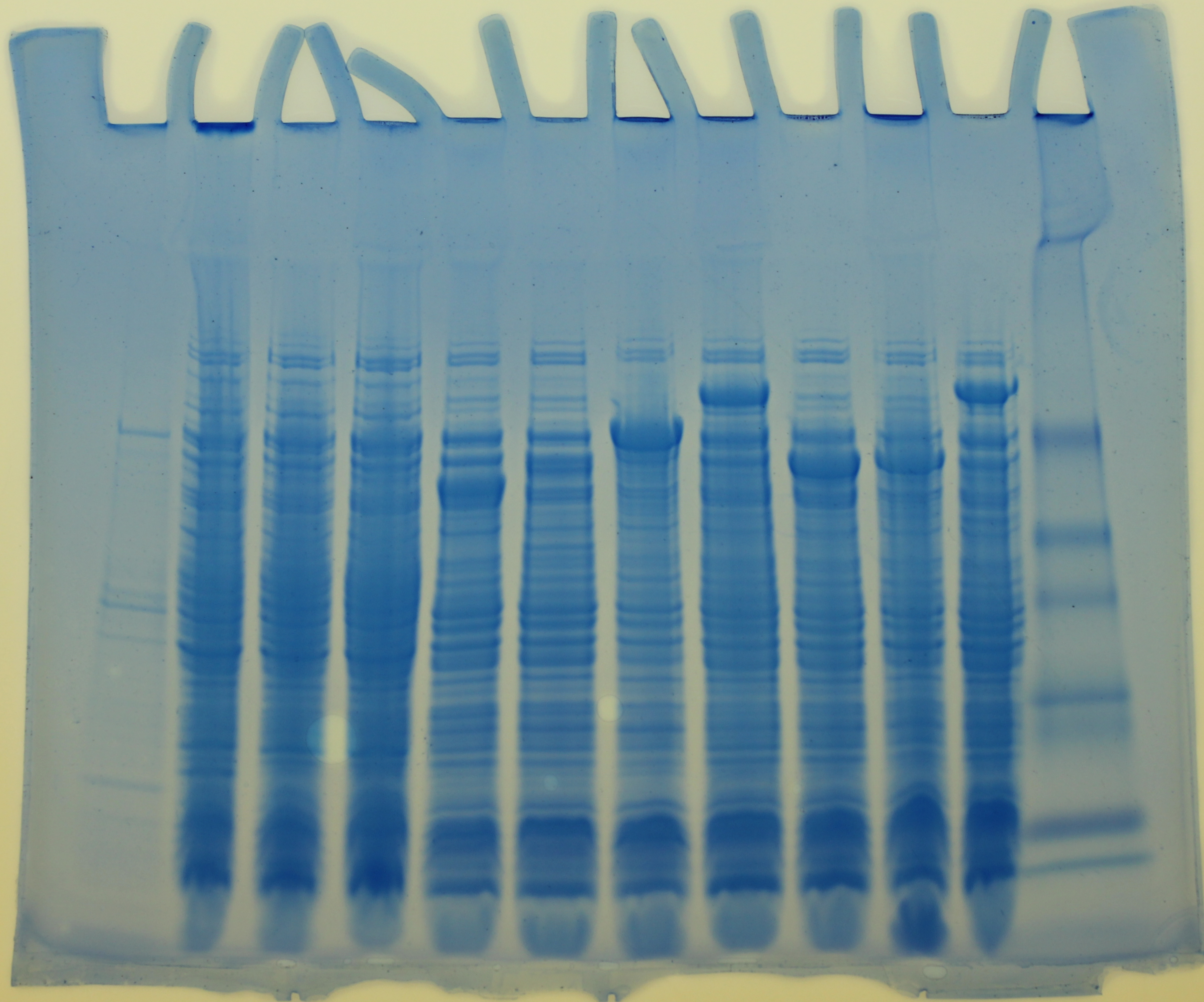

Figure S3\_raw\_image

Supplement: S1 Raw images — (PDF) [file pone.0300503.s008.pdf]
