# Supplementary material for: Searching for new plastic-degrading enzymes from the plastisphere of alpine soils using a metagenomic mining approach
Source: PLoS One. 2024 Apr 5;19(4):e0300503. doi: 10.1371/journal.pone.0300503 (PMC10997104; doi:10.1371/journal.pone.0300503)
Supplement: S1 File — The signal peptides are indicated in red letters. (PDF) [file pone.0300503.s006.pdf]

Candidate gene A
MKYLRLLPISVATTLAMFGVGNAQAQSSITVSGVKYTFCGSENDKCTFSGKGSVVFGSVPPN
SPTTMLSSPRSFTNGVGCYVGAVSSSDPAYGYGKSCWVSLAATTTPPVTTPPVTTPPVTTPP
VTTPPVTTPPVTTPPVTTPPVTTPPVTTPPATTPTPGASAISCGSPTQTAGGTANGLISADT
PTTDGLRIFPNNTAFNIAVTTNSPGADTVVWSVADTNGAIKTQGSFAVKSGVQTATMSCKST
WSGYGSITATLQSHGGTLPNKGTRPMGIATFGVLPNLTSVLGTVTYAHQDQHRFGMQGFNGN
IAALHALGISWTIDDREVSAMEPNGPNTYTPSVNDLDPFYKANPDQMRIVRLDGLPAWDSKT
GQFNDSYYAPSNMTEFQNFMGRVGTDTSLIRAANYPKQQNNYYQVTWEPSVGWADSQANFVA
MYKAAYQGLHSTDPNAIVMGPTNPFPANCDVCTTGYLQTFGALGLWNYIDAVSTHGYWNAGT
YPAHPPELQDSDPNPANQANALDNLMTQLRAVMQAGKPNMKLFVTEAGTSYDPGINYGPTSP
SQNQLFAQAAVGVRSHIITLGGGAQMTTFFYGADYPGETGYGTFFDLNDAQGAWGASNLSPK
PEALAFATLTRVLDGTNTLGRVKGMAAGTYAYAFQQLGNGKVVTAVWAHSNAQWPASGGLYS
QTYSTSYSLQVDAAGASGNVTEIDGYGNVSTVPYTNGKVTLTATEVPQYIVSSNATVAKANA
TVPVGYTGQ

Candidate gene B
MRSAFFIIGLCVALLGCASIQNAPVNEPGNSTGLADRVHLNFEEQGQDEDNLIGMSFSGGGT
RAAAFSFGVLTEMAQTPVRGGRASMLDHLDFISGVSGGAVTAAYYGLRKRAALDDFRERFLL
RNAEEGLQTDLNLGTIGRALAGGINDSRGFPRWLDANLFHGATFAEFREAGRPRVWINASDI
YNRTPFVFGATTFNAMCSDLSKYPLANAVAASAAVPVAFAPVVIQTFPGTCNDPLPAWIIKA
RDDSHAPPMLNSFAKAINRYHDGQMPYIKLLDGGLVDNYGLSGFTIARLSAETPYGPMTPQQ
AVKLRRALFLVVDAKTGVSGNWINSVEGPTGVELVKAAADTAIDASVGASFTAFDRTMADWQ
SSLIKWRCGLSAADRARFGARPGWNCHDLKFFIGRLGFDQLDPARATELEAIPTRFRLPPEQ
VDSVISGGRDALRANPTFRAFAGSL

Candidate gene C
MFGKFSRRHFARLAGFSALGIATAPAKAADGEPKPAADRHAPASFPEGFVWGTATSAYQIEG
AVHEDGRGPSIWDIFAHTPGKIADHSNADRANDHYHRYKEDVGLIKALGVKAYRFSIAWPRV
FPDGNGAPNPRGLDFYDRLVDELLSNGIEPFATLYHWDLPQPLQDKIGGWQSSETSRAFAAY
AGYVAERLSDRVKNIFTVNEAGRFVNFGYGWGIDAPGLKLPPAKLNQVRHNVALGHGLAVQA
IRARGRAGVKVGPAENIAACVPAIDTPENIRAAEIATRELNAGFLGVILEGKYTDGFLEYAG
KDAPKFTADELKIIGSPIDFVGLNIYAPQFYVVAKDRAPGFDVLPFPASFPHMKSEWLRIGP
ETAYWVPRIVAKIWNVDTIYISENGTSSEDKVSADGKVYDLDRVMYLRNYLTQLQRATSEGV
PVRGYFLWSLMDNFEWIFGFEQRFGLYRVDFQTQARIPKLSVSFYRDVIARNAIGS

Candidate gene D
MKFVRALAFFAVFAASPSFADPAVKWGGWDKELFSRATAEKRFVILDLEAVWCHWCHVMEKT
TYSDPKVVELLDSKYLPVRVDQDANPDLSNRYGDWGWPATIVFNSDGTEIAKIRGYIEPERM
QALLKAVIEDPSPGPSVGEAFEIKPSTSTFLTKDQRAELIKNYDESYEDNIGGWGDSQKFID
ADSMDYAMTRAEAGDAVATQRSRQTFDAALALIDPVWGGTFQYSEAGSWAHPHFEKIMSFQA
QYLRQYSQAYAQWKDPKYLTAAANIERYLADFLVSPDGAFYVSQDADLDHYTDGHKYYALAD
ADRRKLGMPRIDKNIYARENGWAISGLAAYYNVTNDPKILAMAQRTAKWVAENRALPGGGFR
HGETDRGGPFLGDTLAMGQAYLDLYAATGNRDWLTEAGKAGDFIGAAFKDDAGGFFTSKTTE
ANVGVFAKPAKLNDDQTQVVRFMNMLNRYFGNDVYRDYASHAMKYLTAASVDAGRPLPGVLL
ADEELAVEPTHMTIVGHKDDPRAQALFATARAFPARYKRLEWLDPREGKLPNPDVEYPDMGD
PAAFACSNRICSYPSFNAEELKATVQQMAKLKPARTALD

Candidate gene E
MDMSHPSLFIPSSKEYEMTVQLTRRDMLKRASGLLAVAAVGGLSIGAEAAEGHQERNPVLVR
NPAFASIDLCLRQAVDNGTVAGVVAMGATQRGLIYEGASGHANPQTRTAMTPDTVFWLLSMT
KAITATACMQLIEQGRLRLDQPAGEILPELRSPQVLDGFDASGQPKLRPARNTITVRHLLTH
TSGFTYSIWSENLSRYEKVTGMPDIGYSMNGAFKAPLAFEPGERWQYGIGMDWVGKLVEAVT
DQSLEVYFREHIFTPLGMSNSGFLISSAQKQRVATMHNRQPDGSLKPAPFEINQRPEFFMGG

GGGFSTPRDYMALLQMLMNGGTYRGERILRADTVATMFQNQIGDLQVTEMKTAQPAWSNSFD
QFPGTPHKWGFSFDINTQPGPHGRSAGSISWAGLLNSYFWVDPVGRVAGTLFTQILPFYDPR
VVDLYGQFEQGLYNGLQHA

Candidate gene F

MNLRALSTVAAATLFASLLSPTSALADDAPAEHWVSAWGTALQAIPQRPDLPALYRAPEIGG
RTVRQIVYPAIDGRHVRLRLSNVYGTAPLVIEGVQVARSASGGAAAIRAGTSRPVTFAGKAG
VTIAPGGQTDSDPVAFDVTAHQPLAVSTYMGAGQKMAAWHRVANQTNYVSTPGNHSSDTDAA
AFRTRFTQFVWLTSVSVDAAPARALVAIGDSITDGMRSTPNANRRWPDALARRLTQKGIDGT
AVVNAGISGNRLLSGSPCYGDALLNRFDRDALRQPGVRAVILMIGINDINFPSMPPRAGLDC
DDPHTPVTADLLLRGYQRLIAQAHQRGVRIYGATLTPASLPPEREAIRTAVNDSIRSSRAFD
GVIDFDQALRDPARPDRLQRRYDSGDHIHPGDAGYAAMSEAVPIDEMGLGKGH

Candidate gene G

LKKYPQKALGWTLAATLPLIVASCGGSDSSTVSDPNLVQTAQGQAKGVAVNGVHAYLGLPYA
APPVGALRWKAPVAAAAYSGVRDASHAGSECVQGSPAATAGSEDCLYMNIYVPGPTTVTTPL
PVLFWIHGGGFINGSGIATDGSALAVKANAIVVTFNYRLNALGFLAHPALAAEDPNGAAGNY
GIMDQAAALTWVQKNIAAFGGDPKNVTIFGDSAGGHSVYVQLASPGSAGLFAKAVAQSGDFS
QVQATLTQAETSGAALASGWGCGTTPSADCLRQLPASATLQGNPNAWYAIVDGKVLPTSTSQ
AFAAGTFNRVPLISGFTQNEGTFFVAAAFDAQGNPVQATNYTNTIMGYLGVPGASTAALYAL
SQYSSPSQALAAALGDYRFVCTALQDGDNLAKFAPAVYMYRFSDPAPYNLGGLTSILPPTTL
NYGTYHSSDLDYWWQLIPTPTANQATLSDAMTAALSSFAHSGNPNTGSTVANWPAYTSATRR
VLDFGYPVSNTYDAYTAHNCSYWFGQPPSQHL

Candidate gene H

MRRTHWRRASPLCLLTLIALTACGGGGGGGPGFLPVFGGNPVSPPAQDSYKAEIRRTAFGVP
HIKADNFEGVGYGYGYAQAQDSLCTLADSFLTYRGERSRYFGADAQSVYAGTLGRPLNLESD
FFHKHVITADTLDAMRAAQPDTLRKLVEGFAAGYNRYVREIKAGGPENAACGKEAWVAPITP
DDIYRRMYHAGLAGGYSNFVSGIAAAVPPSPQVAKLAAGNATMLKTASSTARPAVLPPIQVG
GQKGIGSNMIGFGTTATGDASPLLFGNPHWYWHGPDRLYQAHLTVPGQLNVSGASFPGVPVM
LLGFNDNVAWSHTVSTAKRYSLYQLQLAKDDATSYVRDGQNVKMQPTAITVTVKQPSGSLMQ
VTRTLYRSAYGPLVDLSGIDPSFAWSPSIAFAVRDINSQNYRVWRSWLRWNQAKSLDELVAV
QREEAAVPWVNTVAVGRGSAKAWYADMGAVPNVSDAQIAQCNTDEGRALAALFGGGADAPIV
LDGSRSACDWKDDPDSAQPGAIGPSRLPSLWRDDYVANMNDSYWLANPKAPLTGYPSIMGPA
GTAPVSFRTQLGNRLAQDRLEGTDGYAGDKATVDTVKQMVLNSRAYNAEIFKTQMLDIVCAV
PTISVIGDPLGEGVFPSPRDVDTALACDVLRQWGNTGNIGARGAHIWDEFWSRASQLDAGAL
YAVPFSASDPLHTPRGVKSSAAVDLQQAFGAAVLLVKASPYPLDAARGDYLFTVRGGVKIPL
YGGCVDGYFTHICANNRLDKGGYNMDSDASANGNSYIQIVRFPQGGVEAHTLVSYSVSEDPA
SPHFGDYTQAYGNKQWLRLPFSEGEITGDAAYRSVTVRE

Candidate gene I

MSGLVSGLIVLGLAGGPSAVMAQEPGAAPANASAAPADTQAPPNAAKPSAQANDRAASDANI
NPARRNRAIAARTAATTDTASETKADTLIPVPPETTSVTQHSIRLDGRKIDYTATAGNLLLR
NNTGQAEASVFYVAYTATTKSTATRPVTFLFNGGPGAGSVFLLMGSFGPKRAHTSSPAITAP
APYVLADNPDSLLDTTDLVFIDAPGAGFSRIVGHATGKRFWGVDEDLDAFEHFIERYLSVNQ
RWNSPKYLLGESYGTARAAMLAYRLSQNNIALNGVVLMSSILNSGAHMEGTDLESESYLPTY
AAIAWYHDKIVPKPPSLPAFLDEVRAFASGPYAQALAAGDSLPDSERDAIAARVAHYTGLDV
NYVKQTRLQIWPSRFRKQLLRSESRTVGRYDARSEGIDFDDADGRPDYDPSVTSVSSAFDAA
FHEHLAQDLHFEPKDAYRVFNDEALRQWNWKHRAWWGEQLQLPYAAGDLAEAIRQNPQLRVL
SLNGYFDLATPFFQTEYDLAHMELDPSLRANVQRTYYPTGHMIYLDDAALHLLKSDLVRFYS
GGTAQTPKTGSQ

Control gene Z

MLLKKRWLIFISFLLAFTLLIPTAASASEKHYKPNIALEPIENSEGNEHPIILVHGLGGFGR
DELGGIIKMWGGIHDIEKKLREKGYKVYTAAVGPVSSNRDRAIELYYQIKGGTVDYGEAHAK
KYGHDRYGRTYPGFYPEWGEINPKTGKPNKVHLIGHSMGGQTIRTLAQLLYEGDPEEHKTGG
NDISPLLSGEKQPWLHSVLSISSPHDGSTATYLVNDVIPIIQELVIGAAIFAGNIDQNLYDF
KLDHWGIKKRPGESFHSYVQRVRNSPGWKTKDTANWDLKPEGAYELNRWVKAQPDVYYFSVS
NTQSRRSLLTGYYVPDLFMNPFLHPTAYYIGSKTFRKSNFVLDKTWWENDGLVSVKAMKGPN
IGSNDVIVEYNGTPRKGVWNHLGTMRQFDHLDIIGWGVRDVTSWYEDVARFLYSLPDDY
