# Supplementary material for: Searching for new plastic-degrading enzymes from the plastisphere of alpine soils using a metagenomic mining approach
Source: PLoS One. 2024 Apr 5;19(4):e0300503. doi: 10.1371/journal.pone.0300503 (PMC10997104; doi:10.1371/journal.pone.0300503)
Supplement: S2 File — (PDF) [file pone.0300503.s007.pdf]

Candidate gene A

cagagcagcatcaccgtgagcggtgttaagtataccttctgcggcagcgagaacgacaagtg
caccttcagcggcaaaggtagcgtggtttttggtagcgttccgccgaacagcccgaccacca
tgctgagcagcccgcgtagctttaccaacggtgtgggttgctatgtgggtgctgtgagcagc
agcgacccggcgtacggctatggtaaaagctgctgggttagcctggcggcgaccaccacccc
gccggtgaccaccccgccggttactaccccgccggtgactaccccgccggttaccaccccgc
cggttacaaccccgccggtcactaccccgccggtcacaaccccgccggtcacgaccccgccg
gtcaccaccccgccggcgaccaccccgaccccgggtgcgagcgcgatcagctgcggcagccc
gacccagaccgcgggtggcaccgcgaacggtctgattagcgcggacaccccgaccaccgatg
gtctgcgtatcttcccgaacaacaccgcgtttaacattgcggttaccaccaacagcccgggt
gcggacaccgtggtttggagcgtggcggataccaacggtgcgatcaagacccagggcagctt
cgcggtgaaaagcggcgttcaaaccgcgaccatgagctgcaagagcacctggagcggctatg
gtagcatcaccgcgaccctgcaaagccacggtggcaccctgccgaacaaaggcacccgtccg
atgggtattgcgacctttggcgtgctgccgaacctgaccagcgtgctgggtaccgttaccta
cgcgcaccaggaccaacaccgtttcggcatgcaaggttttaacggtaacattgcggcgctgc
atgcgctgggtatcagctggaccattgacgatcgtgaggtttctgcgatggaaccgaacggc
ccgaacacctataccccgagcgtgaacgacctggacccgttctacaaggcgaacccggatca
gatgcgtatcgttcgtctggacggtctgccggcgtgggatagcaaaaccggccagtttaacg
acagctactatgcgccgagcaacatgaccgagttccaaaactttatgggtcgtgtgggcacc
gataccagcctgattcgtgcggcgaactacccgaagcagcaaaacaactactatcaggttac
ctgggaaccgagcgtgggttgggcggacagccaggcgaacttcgttgcgatgtacaaagcgg
cgtatcaaggtctgcacagcaccgacccgaacgcgatcgttatgggtccgaccaacccgttc
ccggcgaactgcgatgtgtgcaccaccggttatctgcaaacctttggtgcgctgggcctgtg
gaactacattgacgctgtgagcacccacggttattggaacgcgggtacctacccggcgcacc
cgccggagctgcaagacagcgatccgaacccggcgaaccaagcgaacgcgctggacaacctg
atgacccagctgcgtgcggttatgcaagcgggcaagccgaacatgaaactgttcgtgaccga
agcgggcaccagctatgatccgggtatcaactacggcccgaccagcccgagccagaaccaac
tgtttgcgcaagcggcggtgggtgttcgtagccacatcattaccctgggtggcggtgcgcaa
atgaccaccttctttacggtgcggactatccgggcgagaccggctacggtaccttctttga
tctgaacgatgcgcaaggcgcgtggggcgcgagcaacctgagcccgaaaccggaagcgctgg
cgtttgcgaccctgacccgtgttctggatggtaccaacaccctgggtcgtgtgaagggtatg
gcggcgggtacctacgcgtatgcgtttcagcaactgggcaacggtaaagtggttaccgcggt
ttgggcgcatagcaacgcgcaatggccggcgagcggcggtctgtacagccagacctatagca
ccagctacagcctgcaagtggatgcggcgggtgcgagcggtaacgtgaccgagatcgatggc
tatggtaacgtgagcaccgttccgtacaccaacggcaaggttaccctgaccgcgaccgaagt
tccgcagtatattgtgagcagcaacgcgaccgtggcgaaagcgaacgcgaccgtgccggttg
gttacaccggccaa

Candidate gene B

tgcgcgagcatccagaacgcgccggtgaacgaaccgggcaacagcaccggtctggcggaccg
tgttcacctgaacttcgaggaacagggccaagacgaagataacctgattggtatgagcttta
gcggtggcggtacccgtgcggcggcgttcagctttggtgtgctgaccgaaatggcgcagacc
ccggttcgtggcggtcgtgcgagcatgctggaccacctggatttcatcagcggcgtgagcgg
cggtgcggttaccgcggcgtactatggtctgcgtaagcgtgcggcgctggacgatttccgtg
aacgttttctgctgcgtaacgcggaggaaggcctgcaaaccgatctgaacctgggtaccatt
ggtcgtgcgctggcgggcggtattaacgacagccgtggtttcccgcgttggctggatgcgaa
cctgtttcacggcgcgaccttcgcggagtttcgtgaagcgggtcgtccgcgtgtgtggatca
acgcgagcgacatttacaaccgtaccccgttcgtttttggcgcgaccaccttcaacgcgatg
tgcagcgatctgagcaagtatccgctggcgaacgcggtggcggcgagcgcggcggtgccggt
tgcgttcgcgccggtggttatccagacctttccgggtacctgcaacgacccgctgccggcgt
ggatcattaaagcgcgtgacgatagccacgctccgccgatgctgaacagctttgcgaaggcg
atcaaccgttaccacgatggccaaatgccgtatattaaactgctggacggcggtctggtgga
taactacggcctgagcggtttcaccattgcgcgtctgagcgcggagaccccgtatggtccga

tgaccccgcagcaagcggttaagctgcgtcgtgcgctgtttctggtggttgacgcgaaaacc
ggcgtgagcggtaactggatcaacagcgttgagggcccgaccggtgtggaactggttaaagc
ggcggcggacaccgcgattgatgcgagcgttggcgcgagcttcaccgcgtttgaccgtacga
tggcggattggcaaagcagcctgatcaagtggcgttgcggtctgagcgcggcggaccgtgcg
cgttttggtgcgcgtccgggttggaactgccacgatctgaaattctttattggccgtctggg
ttttgaccagctggacccggcgcgtgcgaccgagctggaagcgatcccgacccgttttcgtc
tgccgccggaacaagtggacagcgttattagcggcggtcgtgatgcgctgcgtgcgaacccg
accttccgtgcgtttgcgggtagcctg

Candidate gene C
gcggatggtgaaccgaaaccggcggcggatcgtcatgcgccggcgagcttcccggagggctt
tgtttggggtaccgcgaccagcgcgtaccagattgaaggcgcggtgcatgaagatggtcgtg
gtccgagcatctgggatattttcgcgcacaccccgggcaagattgcggaccacagcaacgcg
gaccgtgcgaacgatcactaccaccgttataaagaagatgttggcctgatcaaggcgctggg
tgtgaaagcgtatcgtttcagcattgcgtggccgcgtgtttttccggatggtaacggtgcgc
cgaacccgcgtggcctggacttctacgatcgtctggtggatgagctgctgagcaacggtatc
gaaccgtttgcgaccctgtatcactgggacctgccgcagccgctgcaagataagattggtgg
ctggcaaagcagcgaaaccagccgtgcgtttgcggcgtacgcgggttatgttgcggaacgtc
tgagcgaccgtgtgaaaaacatctttaccgttaacgaggcgggccgtttcgtgaactttggt
tacggctggggtattgatgcgccgggtctgaagctgccgccggcgaaactgaaccaagtgcg
tcacaacgttgcgctgggccacggtctggcggttcaagcgattcgtgcgcgtggccgcgcgg
gtgtgaaagttggtccggcggagaacattgcggcgtgcgtgccggcgattgacaccccggaa
aacatccgtgcggcggagattgcgacccgtgaactgaacgcgggcttcctgggtgttatcct
ggagggcaagtacaccgacggtttcctggaatatgcgggcaaggacgcgccgaaatttaccg
cggatgaactgaaaatcattggcagcccgatcgatttcgttggtctgaacatttacgcgccg
cagttttatgtggttgcgaaggaccgtgcgccgggctttgatgtgctgccgttcccggcgag
ctttccgcacatgaaaagcgagtggctgcgtattggtccggaaaccgcgtactgggtgccgc
gtatcgttgcgaagatttggaacgttgacaccatctatattagcgagaacggcaccagcagc
gaagacaaagtgagcgcggatggtaaagtttacgacctggatcgtgtgatgtacctgcgtaa
ctatctgacccaactgcaacgtgcgaccagcgagggcgtgccggttcgtggttatttcctgt
ggagcctgatggataactttgagtggatcttcggctttgaacaacgtttcggtctgtaccgt
gtggactttcagacccaagcgcgtattccgaaactgagcgttagcttctatcgtgatgtgat
cgcgcgtaacgcgattggtagc

Candidate gene D
gatccggcggttaagtggggtggctgggacaaagagctgttcagccgtgcgaccgcggaaaa
acgttttgtgatcctggacctggaggcggtttggtgccactggtgccacgtgatggaaaaga
ccacctatagcgatccgaaagtggttgagctgctggacagcaagtacctgccggtgcgtgtt
gaccaagatgcgaacccggatctgagcaaccgttatggcgactggggttggccggcgaccat
tgtgttcaacagcgatggcaccgaaatcgcgaaaattcgtggttacatcgagccggaacgta
tgcaggcgctgctgaaagcggttattgaggacccgagcccgggtccgagcgtgggcgaggcg
ttcgaaatcaaaccgagcaccagcacctttctgaccaaggatcagcgtgcggaactgattaa
aaactacgatgagagctatgaagacaacatcggtggctggggcgacagccaaaagtttattg
acgcggatagcatggattatgcgatgacccgtgcggaagcgggtgatgcggttgcgacccag
cgtagccgtcaaacctttgatgcggcgctggcgctgattgacccggtgtggggtggcacctt
tcagtatagcgaggcgggcagctgggcgcacccgcacttcgaaaaaattatgagctttcagg
cgcaatacctgcgtcagtatagccaagcgtacgcgcaatggaaggacccgaaatatctgacc
gcggcggcgaacatcgaacgttacctggcggatttcctggttagcccggacggtgcgtttta
tgtgagccaggacgcggatctggaccactacaccgatggccacaagtactatgcgctggcgg
atgcggaccgtcgtaagctgggtatgccgcgtatcgacaaaaacatttatgcgcgtgagaac
ggttgggcgatcagcggtctggcggcgtactataacgttaccaacgatccgaagattctggc
gatggcgcaacgtaccgcgaaatgggttgcggaaaaccgtgcgctgccgggtggcggtttcc
gtcacggtgaaaccgatcgtggcggtccgtttctgggtgacaccctggcgatgggtcaggcg

tatctggatctgtatgcggcgaccggtaaccgtgactggctgaccgaagcgggcaaggcggg
tgacttcatcggtgcggcgtttaaagacgatgcgggcggtttctttaccagcaagaccaccg
aggcgaacgtgggcgttttcgcgaagccggcgaaactgaacgacgatcagacccaagtggtt
cgtttcatgaacatgctgaaccgttattttggtaacgatgtttaccgtgactatgcgagcca
cgcgatgaaatacctgaccgcggcgagcgttgatgcgggtcgtccgctgccgggtgtgctgc
tggcggacgaggaactggcggttgaaccgacccacatgaccatcgtgggtcacaaagatgat
ccgcgtgcgcaggcgctgtttgcgaccgcgcgtgcgtttccggcgcgttacaaacgtctgga
gtggctggacccgcgtgagggcaagctgccgaacccggacgttgagtatccggatatgggtg
acccggcggcgtttgcgtgcagcaaccgtatttgcagctacccgagctttaacgcggaggaa
ctgaaagcgaccgtgcagcaaatggcgaagctgaaaccggcgcgtaccgcgctggac

Candidate gene E

gcggagggtcaccaggaacgtaacccggtgctggttcgtaacccggcgttcgcgagcatcga
cctgtgcctgcgtcaagcggttgataacggtaccgtggcgggtgtggttgcgatgggcgcga
cccagcgtggtctgatttatgaaggtgcgagcggtcatgcgaacccgcaaacccgtaccgcg
atgaccccggacaccgtgttctggctgctgagcatgaccaaggcgatcaccgcgaccgcgtg
catgcagctgattgaacaaggtcgtctgcgtctggatcaaccggcgggtgaaatcctgccgg
aactgcgtagcccgcaggttctggacggcttcgatgcgagcggtcaaccgaaactgcgtccg
gcgcgtaacaccatcaccgtgcgtcacctgctgacccacaccagcggttttacctatagcat
ttggagcgagaacctgagccgttacgaaaaggttaccggcatgccggacatcggttatagca
tgaacggcgcgttcaaagcgccgctggcgtttgagccgggtgaacgttggcagtacggcatt
ggtatggactgggttggcaaactggtggaggcggttaccgatcaaagcctggaagtgtattt
ccgtgaacacatctttaccccgctgggtatgagcaacagcggcttcctgattagcagcgcgc
agaagcaacgtgtggcgaccatgcacaaccgtcaaccggatggtagcctgaaaccggcgccg
ttcgagatcaaccaacgtccggagttcttcatgggtggcggtggcggttttagcaccccgcg
tgattatatggcgctgctgcaaatgctgatgaacggcggtacctaccgtggtgagcgtatcc
tgcgtgcggacaccgttgcgaccatgttccagaaccaaattggcgacctgcaagtgaccgaa
atgaagaccgcgcaaccggcgtggagcaacagcttcgaccagtttccgggtaccccgcacaa
atggggcttcagctttgacatcaacacccagccgggtccgcacggtcgtagcgcgggtagca
ttagctgggcgggtctgctgaacagctacttttgggtggacccggttggtcgtgtggcgggt
accctgttcacccagattctgccgttttacgacccgcgtgtggttgatctgtatggccagtt
tgaacaaggcctgtacaacggtctgcaacacgcg

Candidate gene F

gatgatgcgccggcggagcactgggttagcgcgtggggtaccgcgctgcaagcgattccgca
acgtccggacctgccggcgctgtaccgtgcgccggaaattggtggccgtaccgtgcgtcaga
tcgtttatccggcgattgatggtcgtcacgtgcgtctgcgtctgagcaacgtttatggtacc
gcgccgctggtgattgagggtgtgcaagttgcgcgtagcgcgagcggtggtgcggcggcgat
tcgtgcgggtaccagccgtccggttacctttgcgggcaaggcgggtgtgaccattgcgccgg
gtggccagaccgacagcgatccggtggcgtttgacgttaccgcgcaccaaccgctggctgtg
agcacctacatgggtgcgggccagaaaatggcggcgtggcaccgtgttgcgaaccaaaccaa
ctatgtgagcaccccgggtaaccacagcagcgacaccgatgctgcggcgttccgtacccgtt
tcacccagtttgtttggctgaccagcgtgagcgttgatgcggcgccggcgcgtgcgctggtt
gcgatcggcgacagcattaccgatggtatgcgtagcaccccgaacgcgaaccgtcgttggcc
ggatgcgctggcgcgtcgtctgacccagaagggcatcgatggtaccgcggtggttaacgcgg
gcattagcggtaaccgtctgctgagcggcagcccgtgctatggtgatgcgctgctgaaccgt
ttcgatcgtgatgcgctgcgtcaaccgggcgtgcgtgcggttatcctgatgatcggtattaa
cgatattaactttccgagcatgccgccgcgtgcgggtctggactgcgatgatccgcacaccc
cggttaccgcggatctgctgctgcgtggttaccagcgtctgattgcgcaggcgcaccaacgt
ggcgtgcgtatttatggtgcgaccctgaccccggcgagcctgccgccggagcgtgaagcgat
ccgtaccgcggttaacgacagcattcgtagcagccgtgcgttcgatggcgtgatcgactttg
atcaagcgctgcgtgacccggcgcgtccggatcgtctgcaacgtcgttacgatagcggtgat

catattcatccgggtgatgcgggttatgcggcgatgagcgaggcggttccgattgatgaaat
gggtctgggcaaaggtcac

Candidate gene G
tgcggtggcagcgacagcagcaccgtgagcgatccgaacctggttcagaccgcgcagggtca
agcgaagggcgtggcggttaacggtgttcacgcgtacctgggtctgccgtatgcggctccgc
cggtgggtgcgctgcgttggaaagcgccggttgctgcggcggcgtacagcggcgtgcgtgat
gcgagccatgcgggtagcgagtgcgttcagggcagcccggctgcgaccgcgggtagcgaaga
ttgcctgtacatgaacatctatgttccgggtccgaccaccgttaccaccccgctgccggtgc
tgttctggattcacggtggcggttttatcaacggcagcggtattgcgaccgatggtagcgcg
ctggcggtgaaggcgaacgcgatcgtggttaccttcaactaccgtctgaacgcgctgggctt
tctggcgcacccggcgctggcggcggaggacccgaacggtgcggcgggtaactatggtatta
tggatcaggctgcggcgctgacctgggttcaaaagaacatcgcggcgttcggcggtgacccg
aaaaacgtgaccattttggtgatagcgcgggcggtcacagcgtgtatgttcagctggcgag
cccgggcagcgcgggtctgtttgcgaaggcggttgcgcagagcggtgactttagccaggtgc
aagcgaccctgacccaagcggagaccagcggtgcggcgctggcgagcggttggggttgcggc
accaccccgagcgcggactgcctgcgtcagctgccggcgagcgcgaccctgcaaggtaaccc
gaacgcgtggtatgcgatcgtggatggcaaagttctgccgaccagcaccagccaggcgtttg
cggcgggtacctttaaccgtgttccgctgattagcggtttcacccaaaacgaaggcaccttc
tttgttgctgcggcgtttgatgcgcagggtaacccggtgcaagcgaccaactacaccaacac
catcatgggctatctgggtgtgccgggtgcgagcaccgcggcgctgtacgcgctgagccagt
atagcagcccgagccaggcgctggctgcggcgctgggtgactaccgtttcgtttgcaccgcg
ctgcaagacggcgataacctggcgaaattcgcgccggcggtgtacatgtatcgttttagcga
tccggcgccgtacaacctgggcggtctgaccagcatcctgccgccgaccaccctgaactacg
gtacctatcacagcagcgacctggattattggtggcagctgattccgaccccgaccgcgaac
caagcgaccctgagcgatgcgatgaccgcggcgctgagcagctttgcgcacagcggtaaccc
gaacaccggcagcaccgttgcgaactggccggcgtacaccagcgcgacccgtcgtgttctgg
acttcggttatccggttagcaacacctacgatgcgtataccgcgcacaattgcagctactgg
tttggccagccgccgagccaacacctg

Candidate gene H
tgtggtggcggtggcggtggcggtccgggtttcctgccggtgtttggcggtaacccggttag
cccgccggcgcaggacagctataaggcggaaatccgtcgtaccgcgttcggtgtgccgcaca
ttaaagcggataactttgagggtgttggctacggttatggctacgcgcaggcgcaagatagc
ctgtgcaccctggcggacagcttcctgacctatcgtggcgaacgtagccgttactttggtgc
ggatgcgcagagcgtgtatgcgggtaccctgggtcgtccgctgaacctggagagcgatttct
ttcacaagcacgttattaccgcggacaccctggatgcgatgcgcgcggcgcaaccggacacc
ctgcgtaaactggtggaaggcttcgcggcgggttataaccgttacgttcgtgagatcaaagc
gggcggtccggaaaacgcggcgtgcggtaaagaggcgtgggtggcgccgatcaccccggacg
atatttatcgtcgtatgtaccacgcgggtctggcgggcggttacagcaactttgttagcggt
attgcggcggcggtgccgccgagcccgcaggttgcgaagctggcggcgggtaacgcgaccat
gctgaaaaccgcgagcagcaccgcgcgtccggcggtgctgccgccgatccaggttggcggtc
aaaagggtatcggcagcaacatgattggttttggtaccaccgcgaccggtgatgcgagcccg
ctgctgtttggtaacccgcactggtattggcatggtccggatcgtctgtaccaggcgcacct
gaccgtgccgggtcaactgaacgttagcggtgcgagcttcccgggtgtgccggttatgctgc
tgggctttaacgacaacgtggcgtggagccacaccgttagcaccgcgaagcgttatagcctg
taccagctgcaactggcgaaagacgatgcgaccagctacgtgcgtgatggtcagaacgttaa
gatgcaaccgaccgcgattaccgtgaccgttaaacagccgagcggcagcctgatgcaagtga
cccgtaccctgtatcgtagcgcgtacggcccgctggttgacctgagcggtatcgatccgagc
tttgcgtggagcccgagcattgcgtttgcggtgcgtgacattaacagccagaactatcgtgt
ttggcgtagctggctgcgttggaaccaggcgaagagcctggatgaactggtggcggttcaac
gtgaggaagcggcggtgccgtgggttaacaccgtggcggttggtcgtggcagcgcgaaagcg
tggtacgcggatatgggtgcggtgccgaacgttagcgacgcgcagatcgcgcaatgcaacac

cgatgaaggtcgtgcgctggcggcgctgtttggcggtggcgcggatgcgccgattgtgctgg
acggtagccgtagcgcgtgcgattggaaagatgatccggacagcgcgcagccgggtgcgatt
ggcccgagccgtctgccgagcctgtggcgtgacgattatgtggcgaacatgaacgacagcta
ctggctggcgaacccgaaagcgccgctgaccggttatccgagcattatgggtccggcgggta
ccgcgccggttagcttccgtacccagctgggcaaccgtctggcgcaagatcgtctggaaggt
accgacggctatgcgggtgataaggcgaccgtggacaccgttaaacagatggttctgaacag
ccgtgcgtacaacgcggaaatcttcaagacccaaatgctggacattgtgtgcgcggttccga
ccatcagcgtgattggtgatccgctgggcgagggcgtgtttccgagcccgcgtgacgttgat
accgcgctggcgtgcgacgttctgcgtcagtggggcaacaccggtaacatcggcgcgcgtgg
tgcgcacatttgggacgagttctggagccgtgcgagccaactggatgcgggtgcgctgtatg
cggtgccgtttagcgcgagcgacccgctgcacaccccgcgtggtgtgaagagcagcgcggcg
gttgacctgcaacaagcgttcggcgcggcggtgctgctggttaaggcgagcccgtatccgct
ggatgcggcgcgtggtgactacctgttcaccgtgcgtggtggcgttaagatcccgctgtatg
gtggctgcgttgacggctactttacccacatttgcgcgaacaaccgtctggataaaggtggc
tataacatggacagcgatgcgagcgcgaacggtaacagctacatccagattgtgcgttttcc
gcaaggtggcgttgaagcgcacaccctggttagctatagcgttagcgaggacccggcgagcc
cgcactttggtgactatacccaggcgtacggtaacaaacaatggctgcgtctgccgtttagc
gagggcgaaatcaccggtgatgcggcgtaccgtagcgtgaccgttcgtgag

Candidate gene I
caggaaccgggtgcggcgccggcgaacgcgagcgcggcgccggcggatacccaagctccgcc
gaacgcggcgaaaccgagcgcgcaagcgaacgaccgtgcggcgagcgatgcgaacatcaacc
cggcgcgtcgtaaccgtgcgattgcggcgcgtaccgcggcgaccaccgataccgcgagcgag
accaaagcggacaccctgatcccggtgccgccggaaaccaccagcgttacccagcacagcat
ccgtctggacggtcgtaaaattgattataccgcgaccgcgggcaacctgctgctgcgtaaca
acaccggtcaagcggaagcgagcgtgttctacgttgcgtataccgcgaccaccaagagcacc
gcgacccgtccggtgaccttcctgtttaacggtggcccgggtgcgggcagcgttttcctgct
gatgggtagctttggtccgaaacgtgcgcacaccagcagcccggcgatcaccgcgccggcgc
cgtacgtgctggcggataacccggacagcctgctggataccaccgacctggttttattgat
gcgccgggtgcgggttttagccgtattgtgggtcacgcgaccggcaagcgtttctggggcgt
tgacgaggatctggacgcgttcgagcactttattgaacgttacctgagcgtgaaccagcgtt
ggaacagcccgaaatacctgctgggtgaaagctatggcaccgcgcgtgcggcgatgctggcg
tatcgtctgagccaaaacaacatcgcgctgaacggtgtggttctgatgagcagcattctgaa
cagcggtgcgcacatggagggcaccgatctggagagcgaaagctacctgccgacctatgcgg
cgatcgcgtggtaccacgataagattgttccgaaaccgccgagcctgccggcgttcctggat
gaggttcgtgcgttcgcgagcggtccgtatgcgcaagcgctggcggcgggtgatagcctgcc
ggacagcgaacgtgatgcgattgcggcgcgtgtggcgcactacaccggtctggacgtgaact
atgttaagcagacccgtctgcaaatttggccgagccgtttccgtaaacaactgctgcgtagc
gagagccgtaccgttggtcgttacgatgcgcgtagcgaaggcatcgactttgacgatgcgga
tggtcgtccggattatgacccgagcgtgaccagcgttagcagcgcgttcgacgcggcgtttc
acgagcacctggcgcaggacctgcacttcgaaccgaaggatgcgtaccgtgtgtttaacgac
gaagcgctgcgtcaatggaactggaaacaccgtgcgtggtggggcgagcagctgcaactgcc
gtatgcggcgggtgatctggcggaagcgatccgtcagaacccgcaactgcgtgttctgagcc
tgaacggttacttcgacctggcgaccccgttctttcagaccgagtatgatctggcgcacatg
gaactggacccgagcctgcgtgcgaacgtgcaacgtacctactatccgaccggccacatgat
ttacctggacgatgcggcgctgcacctgctgaagagcgacctggttcgtttttatagcggtg
gcaccgcgcagaccccgaaaaccggtagccaa

Control gene Z
catatgagcgagaagcactacaaaccgaacatcgcgctggagccgattgaaaacagcgaggg
taacgaacacccgatcattctggtgcacggtctgggtggcttcggccgtgacgaactgggtg
gcatcattaaaatgtggggtggcatccacgatattgagaagaaactgcgtgaaaaggctac
aaagtgtataccgcggcggtgggtccggttagcagcaaccgtgaccgtgcgatcgagctgta

ctatcagattaagggtggcaccgttgactatggtgaagcgcacgcgaagaaatacggtcacg
atcgttacggccgtacctatccgggttttacccggagtggggcgaaatcaacccgaagacc
ggtaaaccgaacaaggttcacctgatcggccacagcatgggtggccagaccattcgtaccct
ggcgcaactgctgtatgagggtgacccggaggaacacaaaaccggtggcaacgacatcagcc
cgctgctgagcggcgaaaagcagccgtggctgcacagcgtgctgagcatcagcagcccgcac
gatggtagcaccgcgacctacctggtgaacgatgttattccgatcattcaggagctggttat
cggcgcggcgatttcgcgggtaacatcgaccaaaacctgtatgactttaaactggatcact
ggggcattaagaaacgtccgggtgaaagcttccacagctacgtgcaacgtgttcgtaacagc
ccgggctggaagaccaaagacaccgcgaactgggatctgaaaccggagggtgcgtacgaact
gaaccgttgggtgaaggcgcagccggacgtgtactattttagcgttagcaacacccaaagcc
gtcgtagcctgctgaccggctactatgttccggacctgttcatgaacccgtttctgcacccg
accgcgtactatatcggtagcaaaaccttccgtaagagcaactttgtgctggacaaaacctg
gtgggagaacgatggcctggtgagcgttaaagcgatgaagggtccgaacatcggcagcaacg
atgtgattgttgaatataacggcaccccgcgtaagggtgtgtggaaccacctgggtaccatg
cgtcaattcgaccacctggacatcattggttggggcgtgcgtgacgttaccagctggtacga
ggatgttgcgcgttttctgtatagcctgccggacgattactaagatatc
